# Record-large indium-oxo clusters: synthesis, hierarchical assembly, and efficient optical limiting

**DOI:** 10.1039/d6sc00913a

**Published:** 2026-03-16

**Authors:** Xiuzhen Wang, Yi-An Chen, Xiaofeng Yi, Shumei Chen, Jian Zhang

**Affiliations:** a College of Chemistry, Fuzhou University Fuzhou Fujian 350108 People's Republic of China csm@fzu.edu.cn; b State Key Laboratory of Structural Chemistry, Fujian Institute of Research on the Structure of Matter, Chinese Academy of Sciences Fuzhou Fujian 350002 People's Republic of China xfyi@fjirsm.ac.cn

## Abstract

High-nuclearity indium-oxo clusters (InOCs) represent critical molecular models for understanding indium oxide (In_2_O_3_) nanoparticles, yet their rational synthesis remains a formidable challenge. Herein, we report a dual-ligand strategy to access bixbyite-type In_15_-oxo clusters—the largest discrete indium-oxo cores reported to date. Their strategically labile carboxylate sites enable facile functionalization to generate InOC-38, InOC-39, and InOC-40. Notably, the In_15_ core serves as the highest-nuclearity secondary building unit (SBU) within the InOC family, which can be extended into an In_30_ dimer (InOC-41) *via* a cluster-docking strategy or hierarchically assembled into one-dimensional chains (InOC-42) using multidentate 6-hydroxynicotinate linkers. These architectures, featuring π-conjugated ligands, heavy metals, and dense intermolecular interactions, exhibit exceptional optical limiting (OL) performance. InOC-38 and InOC-41 demonstrate record metrics (*T*_min_ = 0.11 and 0.17; *F*_OL_ = 0.275 and 0.408 J cm^−2^), surpassing state-of-the-art cluster-based materials. Furthermore, their processability into flexible transparent films underscores significant practical potential for optical applications.

## Abstract 2

A dual-ligand strategy yields the largest InOC, assembling into dimers and hierarchical one-dimensional chains with record optical limiting performance.

## Graphical abstract

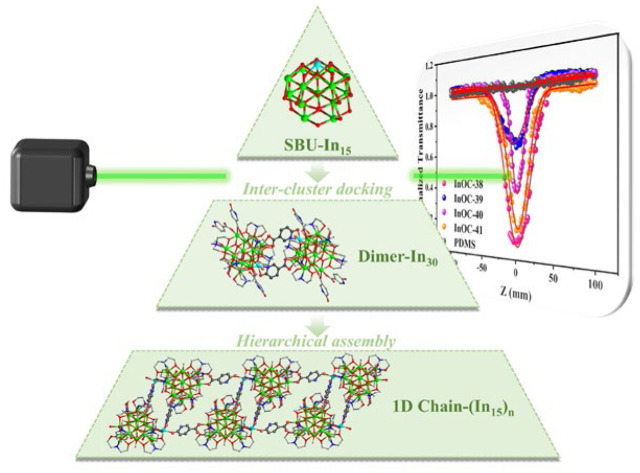

## Introduction

Indium oxide (In_2_O_3_) has emerged as a prominent semiconductor material due to its excellent performance in diverse applications including catalysis, optoelectronics, and sensing.^1–6^ A key obstacle to fully understanding its structure–property relationships, however, is the inherent difficulty in determining its precise atomic-scale arrangement. In contrast, indium-oxo clusters (InOCs)—molecular analogs of In_2_O_3_—exhibit similar In–O compositional characteristics and often form crystalline aggregates with well-defined structures. Unlike bulk or nanostructured In_2_O_3_, InOCs can be characterized with atomic precision *via* single-crystal X-ray diffraction. This makes them an ideal model system for investigating structure–property correlations at the atomic level. Furthermore, the coordination flexibility of In^3+^ centers with various organic ligands leads to considerable structural diversity, encompassing variations in geometry, nuclearity, and overall cluster size. Although research on structurally well-defined InOCs dates back to 1986,^7^ early studies were largely limited to low-nuclearity archetypes such as star,^8,9^ square,^8,10^ square-pyramid,^8,11^ octahedral,^12^ wheel,^13,14^ and bixbyite-like geometries.^15^ To date, the reported nuclearities in this family remain scarce, with notable examples including a sandwich-type In_13_-oxo core and an In_28_ molecular ring assembled from In_7_ secondary building units *via* imidazole linkers.^16^ The synthesis of higher-nuclearity InOCs is an important goal, as larger, nanoscale models could better mimic the behavior of actual In_2_O_3_ nanoparticles. A persistent synthetic challenge, however, lies in controlling the aggregation kinetics of In^3+^ ions. Therefore, developing general and reliable strategies to construct high-nuclearity InOCs and regulate their hierarchical assembly remains an ongoing need.

Optical limiting (OL) materials are critical for protecting sensitive optical devices and human eyes from intense laser pulses,^17^ necessitating attributes such as broadband response, fast nonlinear absorption, and lower optical limiting threshold. Reported OL systems span organic dyes,^18,19^ inorganic semiconductors,^20,21^ carbon-based nanomaterials,^22,23^ and metal–organic complexes.^24,25^ Among these, metal oxo clusters (MOCs) have gained increasing attention due to their well-defined structures, diverse photophysical pathways, and tunable excited state properties.^26,27^ InOCs, in particular, constitute a promising platform for OL applications.^28–30^ The presence of heavy In^3+^ centers can promote intersystem crossing *via* strong spin–orbit coupling and introduce intermediate energy levels, thereby enhancing nonlinear optical responses. Furthermore, their electronic structures can be precisely modulated through ligand engineering—especially with conjugated organic ligands—to improve nonlinear absorption characteristics. Notably, increasing the nuclearity and dimensions of MOCs typically enhances nonlinear absorption, leading to improved OL performance.^31^ Building on this, hierarchical assembly—inspired by strategies observed in biological,^32,33^ nanomaterial,^34,35^ and supramolecular systems^36,37^—offers an additional avenue. Increasing the nuclearity of InOCs and their organization into well-defined hierarchical architectures present a compelling dual strategy to advance OL materials.

**Scheme 1:**
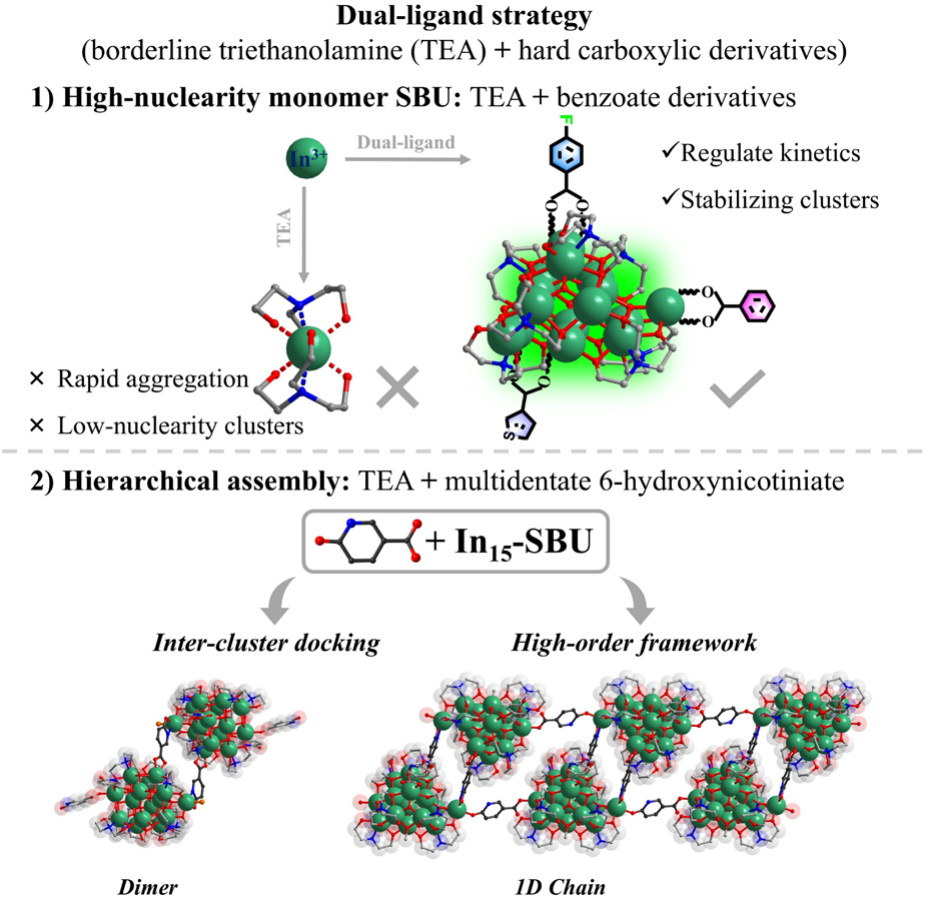
(1) High-nuclearity In_15_-oxo clusters were synthesized *via* a dual-ligand strategy; (2) the In_15_ core can be extended into dimers through inter-cluster docking and hierarchically assembled into one-dimensional chains using 6-hydroxynicotinate linkers.

Guided by the hard and soft acids and bases theory, In^3+^ exhibits strong affinity for nitrogen donors, often leading to rapid aggregation and low-nuclearity clusters. To overcome this, this study develops a dual-ligand strategy, integrating borderline Lewis base triethanolamine and hard aromatic carboxylic derivatives, for constructing high-nuclearity InOCs and their hierarchical assembly with enhanced OL performance (Scheme 1). Triethanolamine promotes controlled nucleation, while aromatic carboxylate derivatives regulate kinetics, stabilizing larger polynuclear species. This approach yielded an unprecedented In_15_-oxo core—the largest InOC building block reported. Functionalization with different benzoate derivatives (InOC-38–40) allows fine-tuning of the OL properties. Moreover, using multidentate 6-hydroxynicotinate as a linker, the In_15_ core was extended into an In_30_ dimer (InOC-41) as the largest InOCs till now *via* a cluster-docking strategy or hierarchically assembled into one-dimensional chains (InOC-42). They are respectively formulated as [In_15_L_4_(HL)_2_(C_6_H_12_O_3_N)_7_(µ_4_-O)_6_(µ_3_-O)_2_(µ_2_-OH)(µ_3_-OH)_1.7_(µ_3_-OCH_3_)_1.3_]·(HL)·(H_2_O)_1.49_·(CH_3_OH)_4_ (HL = benzoate for InOC-38), [In_15_L_4_(HL)_2_(C_6_H_12_O_3_N)_7_(µ_4_-O)_6_(µ_3_-O)_2_(µ_2_-OH)(µ_3_-OH)_2.05_(µ_3_-OCH_3_)_0.95_]·(HL)·(H_2_O)_1.05_·(CH_3_OH)_8_ (HL = 4-fluorobenzoate for InOC-39), [In_15_L_4_(HL)_2_(C_6_H_12_O_3_N)_7_(µ_4_-O)_6_(µ_3_-O)_2_(µ_2_-OH)(µ_3_-OH)_3_]·(HL)·(CH_3_OH)_6_ (HL = 3-thiophenezoate for InOC-40), [In_30_L_2_(HL)_2_(H_2_L)_4_(C_6_H_12_O_3_N)_14_(µ_4_-O)_12_(µ_3_-O)_4_(µ_2_-OH)_2_(µ_3_-OH)_3.144_(µ_3_-OCH_3_)_2.856_Cl_1.62_(NO_3_)_0.38_]·(H_2_O)_1.144_·(CH_3_OH)_20_·(C_6_H_15_NO_3_)_10.8_ (H_2_L = 6-hydroxynicotiniate for InOC-41), and {In_15_L_1_(HL)_2_(H_2_L)_1_(C_6_H_12_O_3_N)_7_(µ_4_-O)_6_(µ_3_-O)_2_(µ_2_-OH)(µ_3_-OH)_1.3_ (µ_3_-OCH_3_)_1.7_·(H_2_L)·(H_2_O)_1.05_·(CH_3_OH)_13_·(C_6_H_15_NO_3_)_6_}_*n*_ (H_2_L = 6-hydroxynicotiniate for InOC-42). Leveraging the heavy-atom effect and intercluster interactions, the clusters were embedded into polydimethylsiloxane (PDMS) films for Z-scan nonlinear optical tests. These InOCs@PDMS composites exhibit strong reverse saturable absorption, highlighting their potential for OL applications.

## Experimental

### Materials and instruments

All of the reagents and solvents employed are purchased commercially and used as received without further treatment. InCl_3_·4H_2_O, In(NO_3_)_3_·*x*H_2_O, benzoic acid, 4-fluorobenzoic acid, and 3-thiophenezoic acid were purchased from Adamas. 6-hydroxynicotinic acid and sodium hydroxide standard solution (1 M) were purchased from Aladdin. Triethanolamine and methanol were bought from Sinopharm Chemical Reagent Beijing. Powder X-ray diffraction (PXRD) data analyses were performed on a Rigaku Mini Flex II diffractometer using Cu Kα radiation (*λ* = 1.54056 Å) under ambient conditions. The Fourier transform infrared (FT-IR) spectra (KBr pellets) were recorded on a Bruker VERTEX 70 instrument over a range of 4000–400 cm^−1^. Thermal stabilities were investigated using a Mettler Toledo TGA/SDTA 851e analyzer with a heating rate of 10 °C min^−1^ under a N_2_ atmosphere. Elemental analyses were measured on a Vario MICRO elemental analyzer instrument. The energy dispersive spectroscopy (EDS) analyses of single crystals were performed on a JEOL JSM6700F field-emission scanning electron microscope equipped with an Oxford INCA system.

### Synthesis method of InOC-38

A mixture of In(NO_3_)_3_·*x*H_2_O (150 mg, 0.5 mmol), benzoic acid (91 mg, 0.75 mmol), sodium hydroxide standard solution (1 M, 8 drops), methanol (5 mL), and triethanolamine (1 mL) was sealed in a 20 mL vial and transferred to an oven at 80 °C for 5 days. When cooled to room temperature, colorless block crystals formed (yield: 94 mg, 73.9% based on In).

### Synthesis method of InOC-39

A mixture of In(NO_3_)_3_·*x*H_2_O (150 mg, 0.5 mmol), 4-fluorobenzoic acid (105 mg, 0.75 mmol), sodium hydroxide standard solution (1 M, 8 drops), methanol (5 mL), and triethanolamine (1 mL) was sealed in a 20 mL vial and transferred to an oven at 80 °C for 5 days. When cooled to room temperature, colorless block crystals formed (yield: 95 mg, 72.0% based on In).

### Synthesis method of InOC-40

A mixture of In(NO_3_)_3_·*x*H_2_O (150 mg, 0.5 mmol), 3-thiophenezoic acid (64 mg, 0.5 mmol), sodium hydroxide standard solution (1 M, 5 drops), methanol (4 mL), and triethanolamine (0.5 mL) was sealed in a 20 mL vial and transferred to an oven at 80 °C for 3 days. When cooled to room temperature, colorless block crystals formed (yield: 91 mg, 71.4% based on In).

### Synthesis method of InOC-41

A mixture of In(NO_3_)_3_·*x*H_2_O (150 mg, 0.5 mmol), InCl_3_·4H_2_O (50 mg, 0.17 mmol), 6-hydroxynicotinic acid (105 mg, 0.75 mmol), sodium hydroxide standard solution (1 M, 8 drops), methanol (5 mL), and triethanolamine (1 mL) was sealed in a 20 mL vial and transferred to an oven at 80 °C for 5 days. When cooled to room temperature, colorless block crystals formed (yield: 80 mg, 50.3% based on In).

### Synthesis method of InOC-42

A mixture of In(NO_3_)_3_·*x*H2O (150 mg, 0.5 mmol), 6-hydroxynicotinic acid (105 mg, 0.75 mmol), sodium hydroxide standard solution (1 M, 8 drops), methanol (5 mL), and triethanolamine (1 mL) was sealed in a 20 mL vial and transferred to an oven at 80 °C for 3 days. When cooled to room temperature, crystals formed (yield: 77 mg, 41.3% based on In).

### Z-scan measurements

The third-order nonlinear optical (NLO) absorption properties of the above sample were investigated using the open-aperture (OA) Z-scan technique. The irradiation light source was a Nd:YAG laser with a repetition rate of 5 Hz. The laser pulse (period, 5 ns; wavelength, 532 nm) was split into two beams with a mirror. The pulse energies at the front and back of the samples were monitored using two energy detectors. All of the measurements were conducted at room temperature. The InOCs@PDMS samples were mounted on a computer-controlled translation stage that shifted each sample along the *z*-axis.

### Calculation of the nonlinear optical parameters

The relationship of the sample transmission and input fluence can be plotted from the open-aperture *Z*-scan curve. From the input laser pulse energy *E*_in_ and beam radius *ω*(*z*), the light fluence *F*_in_(*z*) at any position can be obtained.

*F*_in_(*z*) is defined as:w
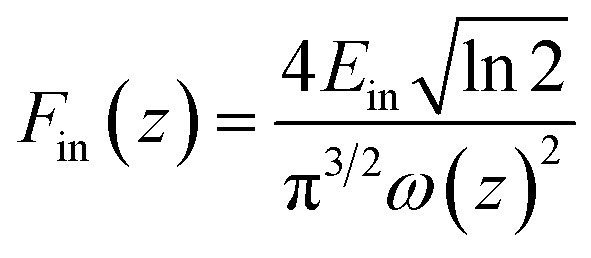
here *ω*(*z*) is defined as:w
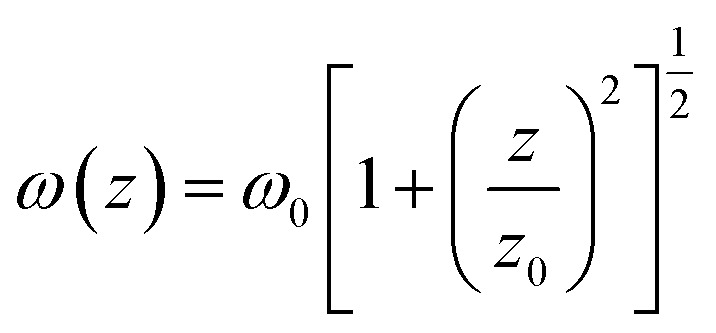
here *ω*_0_ and *z*_0_ are the light beam radius and the Rayleigh range, respectively, and *z*_0_ is defined as:w
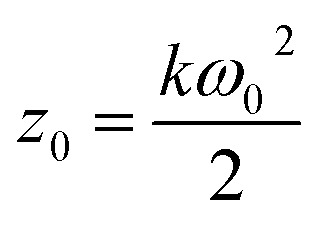
here *k* is defined as:
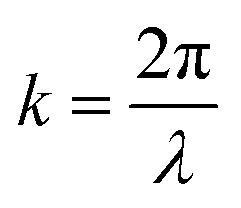


The equation fits for the nonlinear adsorption coefficient *β* as follows:1
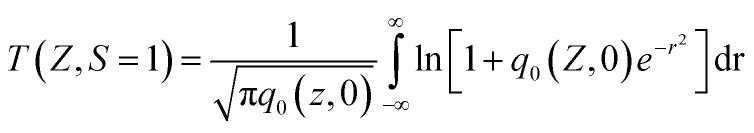

2 *q*_0_(*Z*,0) = *βI*_0_*L*_eff_

3
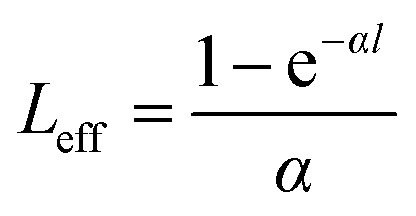
In these equations, *I*_0_ is the on-axis peak intensity at the focus (*Z* = 0), *L*_eff_ is the effective thickness of the sample, *α* is the linear absorption coefficient, and *l* is the sample thickness.

## Results and discussion

### Structural description and characterization

**Fig. 1:**
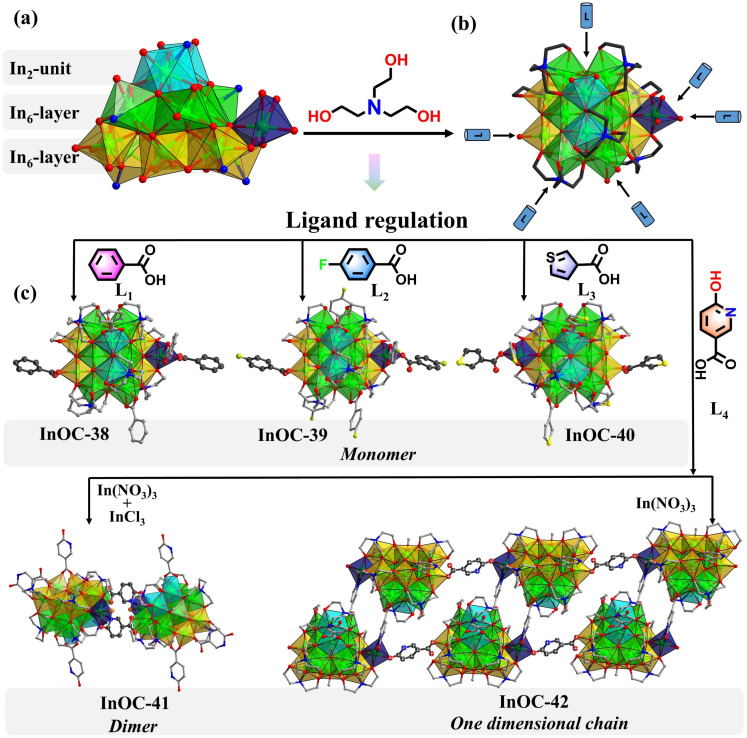
(a) Polyhedral view of the In_15_ core. (b) The In_15_ core was stabilized by triethanolamines and carboxylic derivatives. (c) The structures of InOC38 to InOC-42. Atom color code: In, bright green; Cl, orange; F, green; S, yellow; C, gray; O, red; N, blue. Polyhedron color code: sky blue, In_2_ unit; bright green, In_6_ layer; earthy yellow, another In_6_ layer; dark blue, isolated In center.

The solvothermal reaction of In(NO_3_)_3_ with triethanolamine and benzoic acid in sodium hydroxide standard solution and methanol afforded colorless crystals of InOC-38. Single-crystal X-ray diffraction analysis reveals that the compound features an In_15_-oxo core, whose In–O connectivity resembles that of bixbyite-type In_2_O_3_, the 15 In^3+^ ions are interconnected by six µ_4_-O bridges. Structurally, the core can be viewed as two nearly coplanar In_6_ units linked vertically through four µ_4_-O atoms, supplemented by an In_2_ unit and an isolated In center coordinated at the top and side, respectively, thereby completing the In_15_-oxo architecture (Fig. 1a). The core is stabilized externally by seven triethanolamine ligands and six benzoates (Fig. 1b), with intramolecular hydrogen-bonding interactions further enhancing its stability (Fig. S11). Notably, the carboxylate sites exhibit dynamic exchange behavior, providing a tunable platform for modulating OL properties. To date, this In_15_-oxo unit is the largest reported InOC building block.

To systematically probe how ligand conjugation and substituents influence intramolecular charge transfer—key factors governing OL performance—we replaced benzoic acid with 4-fluorobenzoic acid (bearing a strong electron-withdrawing fluorine substituent) and thiophene-3-carboxylic acid (featuring a weaker conjugation system). The corresponding clusters InOC-39 and InOC-40 were synthesized under identical conditions (Fig. 1c). All three compounds possess an identical In_15_-oxo core, differing only in the peripheral carboxylate ligands. This provides an ideal model system for investigating ligand-dependent effects on OL behavior.

The structural flexibility and external ligand adaptability highlight the potential of the In_15_-oxo core as a versatile modular unit for constructing advanced cluster-based functional materials. Multidentate bridging ligands, in particular, can link discrete oxo-cluster units into higher-nuclearity or hierarchical assemblies. Thus, 6-hydroxynicotinic acid (H_2_NA) was selected as an ideal linker for two reasons: (1) its hydroxypyridine group readily coordinates to indium *via* the nitrogen site, and (2) its para-oriented bidentate site of hydroxypyridine allows chelation analogous to carboxylates, promoting structural expansion. Following this design, reaction with mixed InCl_3_/In(NO_3_)_3_ in methanol produced InOC-41, an In_30_-oxo dimer representing the highest nuclearity InOC reported. Its structure comprises two In_15_-oxo subunits bridged by two NA ligands. Compared to the monomeric In_15_-oxo clusters (InOC-38–40), InOC-41 retains the same In_15_-oxo core, the principal difference lies in the coordination environment of the side isolated In site highlighted in a navy polyhedron, where two original carboxylates are replaced by one chloride ion and one NA ligand from a neighboring In_15_-oxo unit. Importantly, the coordinated chloride on the side In site is potentially replaceable, offering a clear structural basis for further expansion into higher-dimensional architectures *via* ligand substitution.

To verify this hypothesis, we removed InCl_3_ and used only In(NO_3_)_3_ as the indium source, exploiting the weaker coordination ability of nitrate ions, which are more readily displaced. This change effectively avoided competitive coordination from chloride and promoted substitution of the chloride site by the hydroxypyridine moiety. As anticipated, the reaction successfully yielded the one-dimensional chain compound InOC-42, demonstrating controlled structural evolution from a discrete In_30_-oxo dimer to an extended 1D architecture.

The structural characterization confirms the successful synthesis of phase-pure InOC-38–42, as evidenced by the strong agreement between experimental and simulated powder X-ray diffraction (PXRD) patterns (Fig. S17–S21). Thermogravimetric analysis (TGA) reveals their good thermal stability (Fig. S27–S31). Solid-state UV-vis spectroscopy yields optical band gaps of 4.26 eV (InOC-38), 4.34 eV (InOC-39), 4.40 eV (InOC-40), 3.65 eV (InOC-41) and 3.59 eV (InOC-42) (Fig. S32–S36). The wide bandgaps for InOC-38–42 are consistent with their colorless and transparent crystal appearance. This established material integrity provides a solid basis for exploring OL behavior and structure–property relationships.

### Optical limiting performance

**Fig. 2:**
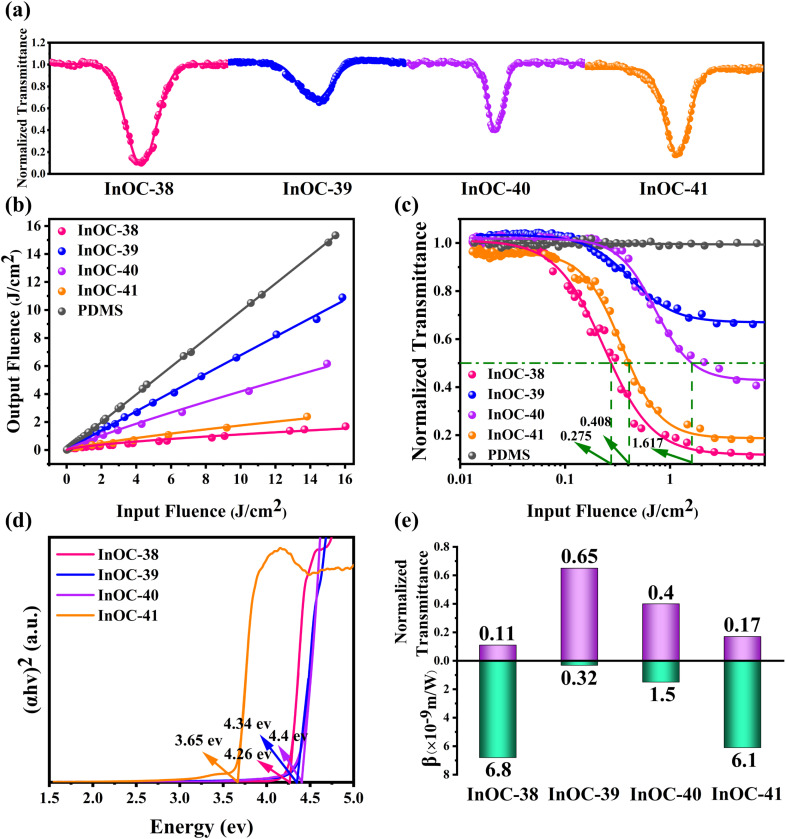
(a) Open-aperture Z-scan plots of PDMS and InOC-38 to InOC-41. (b) Curves of output fluence *versus* input fluence for PDMS and InOC-38 to InOC-41. (c) Variation in the normalized transmittance as a function of input fluence for PDMS and InOC-38 to InOC-41. (d) Optical band gaps determined from solid-state UV-vis spectra for InOC-38 to InOC-41. (e) Comparison of the nonlinear transmittance and nonlinear absorption coefficients *β* of InOC-38 to InOC-41.

Given the presence of heavy metals, rich intermolecular interactions, and the transparent crystal appearance of these materials, their OL performance was systematically evaluated. InOC-38–41 were uniformly dispersed in polydimethylsiloxane (PDMS)—a chemically inert, flexible, and transparent matrix with negligible intrinsic nonlinear optical response—to form InOCs@PDMS composite films. Third-order nonlinear optical properties were investigated under 532 nm nanosecond laser pulses using an open-aperture Z-scan technique. All four composites exhibited reverse saturable absorption (RSA), confirming typical OL behavior. At an input energy of 130 µJ, the minimum normalized transmittances (*T*_min_) at the focal point were 0.11, 0.65, 0.40, and 0.17 for InOC-38–41, respectively (Fig. 2a). The output fluence increased linearly at low input levels but deviated at higher fluences, characteristic of effective OL (Fig. 2b). For quantitative comparison, nonlinear absorption coefficients *β* were derived as 6.8 × 10^−9^, 0.32 × 10^−9^, 1.5 × 10^−9^, and 6.1 × 10^−9^ m W^−1^, respectively (Fig. 2e). The OL thresholds (*F*_OL_, input fluence at 50% transmittance) were determined to be as low as 0.275 J cm^−2^ for InOC-38 and 0.408 J cm^−2^ for InOC-41, while InOC-40 showed a higher threshold of 1.617 J cm^−2^ (Fig. 2c). Repeated scans confirmed excellent reproducibility (Fig. S42–S45). Notably, InOC-38 and InOC-41 demonstrated superior performance, combining high *β* values (6.8 × 10^−9^ and 6.1 × 10^−9^ m W^−1^), low *T*_min_ (0.11 and 0.17), and low F_OL_ (0.275 and 0.408 J cm^−2^), significantly outperforming InOC-39 and InOC-40. These characteristics surpass many reported OL materials, ranking them among the top-performing candidates (Table S8). These results demonstrate the strong potential of these indium-based complexes, particularly InOC-38 and InOC-41, for practical optical limiting applications.

**Fig. 3:**
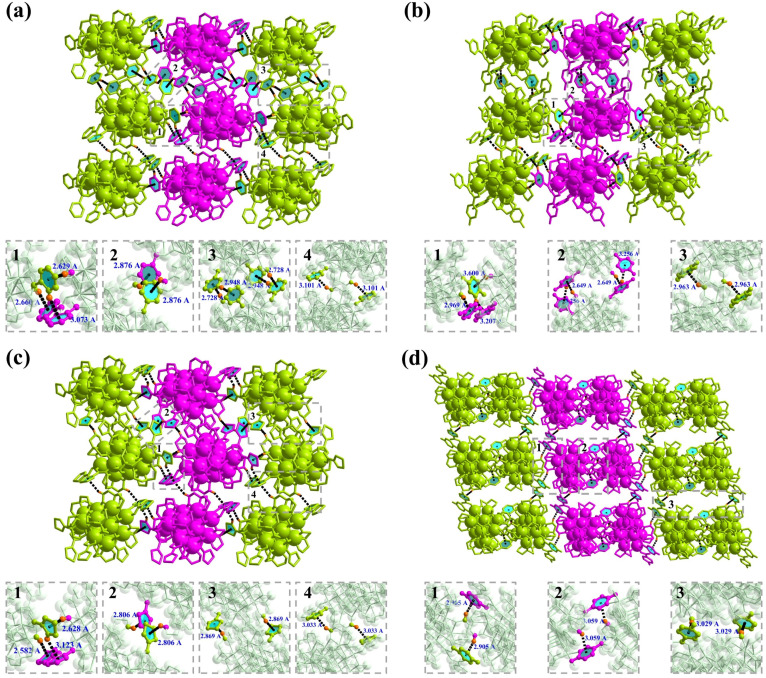
(a–d) Intermolecular C–H⋯π interactions in InOC-38 to InOC-41.

The variations in OL performance among InOC-38–41 stem from the synergistic interplay of five key structural factors: heavy atom effects promoting spin–orbit coupling and triplet state population, reduced optical bandgaps facilitating electron excitation, abundant intermolecular/intramolecular interactions enhancing intersystem crossing, tight molecular packing strengthening electronic coupling, and π-conjugation improving electron delocalization. Specifically, InOC-38 demonstrates the strongest overall OL response, which correlates with its compact molecular packing, effective π-conjugation *via* the benzoate moiety, and the most pronounced C–H⋯π (7 groups, 2.629–3.101 Å, Fig. 3a) and other intermolecular interactions (Fig. S13). In contrast, InOC-41—although featuring the highest heavy-atom content and the smallest bandgap (3.65 eV) (Fig. 2d)—exhibits weaker performance due to its looser packing (Fig. S12), which diminishes intermolecular electronic coupling. Meanwhile, InOC-40 displays limited π-conjugation *via* the 3-thiophenezoate ligand, while InOC-39 has the weakest C–H⋯π contacts, both leading to inferior OL activity. Consequently, the OL efficacy follows the trend InOC-38 > InOC-41 > InOC-40 > InOC-39, which aligns well with the experimentally determined nonlinear absorption coefficients *β*. This correlation confirms a clear structure–property relationship and suggests that high-nuclearity InOCs functionalized with strongly conjugated ligands, tight packing, and strong intermolecular interactions represent an effective strategy for enhancing OL performance.

## Conclusions

We report a dual-ligand strategy employing triethanolamine as a borderline Lewis base to direct controlled nucleation, combined with aromatic carboxylates to modulate reaction kinetics. This approach enabled the isolation of an unprecedented In_15_-oxo core—the largest discrete InOC building block to date. Subsequent functionalization with benzoate derivatives (InOC 38–40) allowed precise tuning of OL properties, while utilizing multidentate 6-hydroxynicotinate as linkers extended the In_15_-oxo core into an In_30_-oxo dimer (InOC-41) *via* a cluster docking approach or hierarchically assembled one-dimensional chains (InOC-42). Benefiting from compact molecular packing, pronounced intermolecular interactions, and high heavy-atom content, InOC-38 and InOC-41 exhibit exceptional OL performance (*T*_min_ = 0.11 and 0.17; *F*_OL_ = 0.275 and 0.408 J cm^−2^). These metrics surpass those of numerous reported materials, ranking them among the top-performing OL systems. This work not only establishes a versatile route to high-nuclearity InOCs and their hierarchical architectures but also demonstrates that supramolecular engineering integrated with cluster expansion offers a powerful design paradigm for advanced nonlinear optical materials.

## Author contributions

All authors contributed extensively to the work presented in this paper. X. -F. Yi, J. Zhang and S. -M. Chen conceived the research project. X. -Z. Wang performed the synthesis, characterization and third-order nonlinear optics studies. Y. -A. Chen assisted with the data collection. X. -F. Yi and X. -Z. Wang wrote the manuscript and SI, with input from the other authors.

## Conflicts of interest

There are no conflicts to declare.

## Supplementary Material

SC-OLF-D6SC00913A-s001

SC-OLF-D6SC00913A-s002

## Data Availability

CCDC 2498685–2498688 for InOC-38 to InOC-41 and 2518939 for InOC-42 contain the supplementary crystallographic data for this paper.^38*a*–*e*^ The data supporting this article have been included as part of the supplementary information (SI). Supplementary information: addition experimental details, general characterization and additional figures. See DOI: https://doi.org/10.1039/d6sc00913a.

## References

[cit1] Qiu C., Sun J., Li M., Mao C., Song R., Zhang Z., Perovic D. D., Howe J. Y., Wang L., Ozin G. A. (2024). J. Am. Chem. Soc..

[cit2] Wulan B., Cao X., Tan D., Ma J., Zhang J. (2022). Adv. Funct. Mater..

[cit3] Zhang N., Cui M., Zhou J., Shao Z., Gao X., Liu J., Sun R., Zhang Y., Li W., Li X., Yao J., Gao F., Feng W. (2024). ACS Appl. Mater. Interfaces.

[cit4] He B., He G., Fu C., Jiang S., Fortunato E., Martins R., Wang S. (2024). Adv. Funct. Mater..

[cit5] Park S., Kim M., Lim Y., Oh D., Ahn J., Park C., Woo S., Jung W., Kim J., Kim I. D. (2024). Adv. Mater..

[cit6] Mo X., Zhu C., Zhang Z., Yan X., Han C., Li J., Attfield J. P., Yang M. (2024). Adv. Mater..

[cit7] Wieghardt K., Kleine-Boymann M., Nuber B., Weisslb J. (1986). Inorg. Chem..

[cit8] Chitsaz S., Neumüller B. (2001). Z. Anorg. Allg. Chem..

[cit9] Mishra S., Daniele S., Petit S., Jeanneau E., Rolland M. (2009). Dalton Trans..

[cit10] Peckermann I., Raabe G., Spaniol T. P., Okuda J. (2011). Chem. Commun..

[cit11] Bradley D. C., Chudzynska H., Frigo D. M., Hursthouse M. B., Mazid M. A. (1988). J. Chem. Soc., Chem. Commun..

[cit12] Hegetschweiler K., Ghisletta M., Fässler T. F., Nesper R. (1993). Angew Chem. Int. Ed. Engl..

[cit13] Saalfrank R. W., Deutscher C., Maid H., Ako A. M., Sperner S., Nakajima T., Bauer W., Hampel F., Heß B. A., van Eikema Hommes N. J. R., Puchta R., Heinemann F. W. (2004). Chem. Eur J..

[cit14] Mishra S., Mendez V., Jeanneau E., Caps V., Daniele S. (2013). Eur. J. Inorg. Chem..

[cit15] Yi X., Wang D., Li F., Zhang J., Zhang L. (2021). Chem. Sci..

[cit16] Zhang R., Lan J., Wang F., Chen S., Zhang J. (2024). Chem. Sci..

[cit17] Zhou G.-J., Wong W.-Y. (2011). Chem. Soc. Rev..

[cit18] Han B., Liang B., Zhang E., Li J., Li Y., Zhang Q., Xie Z., Wang H., Jiang J. (2024). Adv. Funct. Mater..

[cit19] Zhao F., Zhang G., Xie W., Kong X., Duan X., Fu Y., Zhang J., Gao G., Zhu T., Hao J., Li H., Dong R. (2024). Small Struct..

[cit20] Yang Y., Xiao Y., Li B., Chen Y.-G., Guo P., Zhang B., Zhang X.-M. (2023). J. Am. Chem. Soc..

[cit21] Alice Noble A., Joe I. H., Nazar S. (2024). Carbon.

[cit22] Li J., Chen M., Hou S., Zhao L., Zhang T., Jiang A., Li H., Hao J. (2022). Carbon.

[cit23] Ali I., Tan J., Amir M., Musaa Khan M., Shahzada Z., Jamil Y., Wang Y., Fazal Y., Chen J., Shen Z. (2026). Carbon.

[cit24] Jin Z.-B., Zhou G., Han Y., Huang Z., Gu Z.-G., Zhang J. (2024). J. Am. Chem. Soc..

[cit25] Wang Z., Yan Y., Li Q. H., Zhang J. (2025). Aggregate.

[cit26] Gao M.-Y., Wang Z., Li Q.-H., Li D., Sun Y., Andaloussi Y. H., Ma C., Deng C., Zhang J., Zhang L. (2022). J. Am. Chem. Soc..

[cit27] Luo M.-B., Lai H.-D., Huang S.-L., Zhang J., Lin Q. (2024). J. Am. Chem. Soc..

[cit28] Sun X., Yi X., Zhang J., Zhang L. (2023). Chem. Mater..

[cit29] Song Y., Li F., Chen G.-H., Wu J., Yi X., Zhang J. (2025). J. Solid State Chem..

[cit30] Wu J., liu D., Yi X., Zhang J. (2025). Sci. China Chem..

[cit31] Li Y., Zheng C., Wang S. T., Liu Y. J., Fang W. H., Zhang J. (2022). Angew. Chem., Int. Ed..

[cit32] Zhao Y., Liu Z., Qin Z., Wen Q., Du J., Ren X. Y., Chen C. Q., Peng X., Kortz U., Yang P. (2025). Angew. Chem., Int. Ed..

[cit33] Nguyen A. K., Molley T. G., Kardia E., Ganda S., Chakraborty S., Wong S. L., Ruan J., Yee B. E., Mata J., Vijayan A., Kumar N., Tilley R. D., Waters S. A., Kilian K. A. (2023). Nat. Commun..

[cit34] Zhuo H., Dong X., Liu Q., Hong L., Zhang Z., Long S., Zhai W. (2025). Nat. Commun..

[cit35] Bi Y., Cheng C., Zhang Z., Liu R., Wei J., Yang Z. (2023). J. Am. Chem. Soc..

[cit36] Zhang H., Bai F., Qian R., Zhou Y., Zhang L., Sun X. (2025). Coord. Chem. Rev..

[cit37] Song Q., Liu Z., Li J., Sun Y., Ge Y., Dai X. Y. (2024). Adv. Mater..

[cit38] (a) CCDC 2498685: Experimental Crystal Structure Determination, 2026, 10.5517/ccdc.csd.cc2pw2rf

