# Supplementary material for: Record-large indium-oxo clusters: synthesis, hierarchical assembly, and efficient optical limiting
Source: Chem Sci. 2026 Mar 16;17(18):9256–63. doi: 10.1039/d6sc00913a (PMC13006799; doi:10.1039/d6sc00913a)
Supplement: SC-017-D6SC00913A-s001 [file SC-017-D6SC00913A-s001.pdf]

Supplementary Information (SI) for Chemical Science.
This journal is © The Royal Society of Chemistry 2026

Supporting Information

Record-Large Indium-Oxo Clusters: Synthesis, Hierarchical Assembly, and Efficient Optical Limiting

Xiuzhen Wang,[a,b] Yi-An Chen,[a,b] Xiaofeng Yi,[b]* Shumei Chen,[a]* and Jian Zhang,[b]

[a]College of Chemistry, Fuzhou University, Fuzhou, Fujian, 350108.

[b]State Key Laboratory of Structural Chemistry, Fujian Institute of Research on the Structure of Matter, Chinese Academy of Sciences, Fuzhou, Fujian 350002.

*Corresponding Author(s): Xiaofeng Yi: xfyi@fjirsm.ac.cn; Shumei Chen: csm@fzu.edu.cn;

Content

I.Single-Crystal X-ray diffraction S1
II.Bond valence sum calculations S4
III.Additional structural pictures S6
IV.Supramolecular interaction analysis S9
V.Powder-XRD S14
VI.The Energy Dispersive X-Ray Spectroscopy (EDS) spectra S17
VII.Thermogravimetrical analysis (TGA) S19
VIII.Solid-state UV absorption spectra S22
IX.IR spectra S25
X.The repeatability and stability of Z-scan measurements S28
XI.Long-term stability testing. S33
XII.Uniformity of InOCs@PDMS film S34
Reference S35

I. Single-Crystal X-ray diffraction

The structural data of **InOC-38、InOC-39** were collected on a Synergy-R-Mo diffractometer equipped with a graphite-monochromated Mo-Kα radiation source (λ = 0.71073 Å) at 100 K. Crystallographic data for **InOC-40 and InOC-42** were collected on a Supernova single-crystal diffractometer equipped with graphite-monochromatized Cu-Kα radiation (λ= 1.54184 Å) at 100 K. The single-crystal diffraction data of **InOC-41** was collected on Hybrid Pixel Array detector equipped with Ga-Kα radiation (λ= 1.3405 Å) at 100 K. Using Olex2, the crystal structures were determined by direct methods with ShelxT and refined by the full-matrix least-squares method based on F^2 with the SHELXL 2018. Highly disordered solvent regions were present in the structure that could not be modeled sensibly. These solvent contributions were treated using the solvent mask routine (smtbx.mask) as implemented in Olex2. Further details can be found in the CIF file via the _smtbx_masks_special_details and related data items. Non-hydrogen atoms were refined anisotropically. All hydrogen atoms on C and O were bonded by theory. The obtained crystallographic data for **InOC-38** to **InOC-42** are summarized in **Table S1** and **Table S2**.

Table S1 Crystal data and structure refinement for **InOC- 38 to InOC-40.**

Compound	InOC-38	InOC-39	InOC-40
**Crastal formula**	$C_{96.3}H_{147.58}In_{15}N_7O_{52.49}$	$C_{99.95}H_{155}F_7In_{15}N_7O_{56.05}$	$C_{83}H_{136}In_{15}N_7O_{53}S_7$
**Formula weight**	3965.53	4206.80	4026.70
**Crystal system**	triclinic	triclinic	triclinic
**Space group**	P-1	P-1	P-1
**a/Å**	15.6948(3)	15.3911(2)	15.5466(2)
**b/Å**	17.2125(2)	17.1544(2)	17.0764(2)
**c/Å**	24.8215(4)	26.9239(3)	24.2512(2)
**α/°**	76.0650(10)	105.2860(10)	75.8810(10)
**β/°**	87.6540(10)	96.3120(10)	87.5360(10)
**γ/°**	70.4950(10)	103.5060(10)	69.6850(10)
**Volume/Å^3**	6128.91(17)	6555.04(14)	5849.19(12)
**Z**	2	2	2
**ρ_{calc}g/cm^3**	2.149	2.131	2.286
**μ/mm^{-1}**	2.855	2.685	25.120
**F(000)**	3859.0	4100.0	3908.0
**GOF on F^2**	1.029	1.011	1.058
**R_1^a/wR_2^b[I>=2(I)]**	0.0284/0.0599	0.0368/0.0799	0.0383/ 0.0967
**R_1^a/wR_2^b [all data]**	0.0424/0.0637	0.0557/0.0886	0.0463/ 0.1023

$^a R_1 = \Sigma||F_0| - |F_c||/\Sigma|F_0|$. $^b wR_2 = \{\Sigma[w(F_0^2 - F_c^2)_2]/\Sigma[w(F_0^2)_2]\}^{1/2}$.

Table S2 Crystal data and structure refinement for **InOC-41 and InOC-42.**

Compound	InOC-41	InOC-42
Crystal formula	$C_{219.66}H_{456}Cl_{1.62}In_{30}N_{33.18}O_{144.68}$	$C_{122.7}H_{255}In_{15}N_{18}O_{79.3}$
Formula weight	9379.51	4973.93
Crystal system	triclinic	triclinic
Space group	P-1	P-1
a/Å	15.3165(2)	19.5767(4)
b/Å	22.1037(3)	20.3062(4)
c/Å	24.5398(3)	22.4453(5)
α/°	106.7630(10)	111.330(2)
β/°	102.0270(10)	97.119(2)
γ/°	109.5980(10)	104.915(2)
Volume/Å^3	7052.37(17)	7793.3(3)
Z	1	2
ρ_{calc}g/cm^3	2.208	2.120
μ/mm^{-1}	13.912	18.308
F(000)	4661.0	4973.0
GOF on F^2	1.048	1.020
R_1^a/wR_2^b[I>=2(I)]	0.0316/0.0850	0.0756/0.2051
R_1^a/wR_2^b [all data]	0.0402/0.0895	0.0952/0.2191

$^a R_1 = \Sigma||F_0| - |F_c||/\Sigma|F_0|$. $^b wR_2 = \{\Sigma[w(F_0^2 - F_c^2)_2]/\Sigma[w(F_0^2)_2]\}^{1/2}$.

II. Bond valence sum calculations

Bond valence sum calculations are performed on **InOC-38 (Table S3), InOC-39 (Table S4), InOC-40 (Table S5), InOC-41 (Table S6) and InOC-42 (Table S7).** the BVS values are consistent with the oxidation state +III for all In centers.

Table S3 Bond valence sum values for oxygen atoms in **InOC-38**.

In1	2.97	In2	2.96	In3	3.04
In4	2.91	In5	3.00	In6	3.00
In7	2.90	In8	2.93	In9	3.15
In10	2.89	In11	3.10	In12	2.90
In13	2.99	In14	2.87	In15	2.91
O1	−1.96	O2	−2.02	O3	−1.96
O4	−1.98	O5	−2.02	O6	−1.90
O7	−1.94	O8	−1.72	O	−1.05
O12	−1.12	O18	−1.08		

Table S4 Bond valence sum values for oxygen atoms in **InOC-39**.

In1	3.02	In2	3.02	In3	3.03
In4	3.03	In5	3.07	In6	3.02
In7	3.00	In8	2.92	In9	2.92
In10	3.17	In11	3.01	In12	2.93
In13	2.86	In14	2.94	In15	2.95
O1	−2.03	O2	−2.00	O3	−1.98
O4	−1.89	O6	−2.03	O11	−1.76
O12	−1.98	O20	−1.96	O10	−1.07
O19	−1.13	O22	−1.13		

Table S5 Bond valence sum values for oxygen atoms in **InOC-40**.

In1	2.95	In2	3.01	In3	3.02
In4	3.14	In5	3.02	In6	3.03
In7	2.92	In8	2.90	In9	2.92
In10	2.93	In11	2.90	In12	3.08
In13	2.99	In14	2.85	In15	2.92
O1	−1.97	O2	−1.72	O3	−1.91
O4	−1.94	O7	−2.01	O9	−1.94
O23	−1.96	O46	−2.02	O13	−1.15
O19	−1.11	O12	−1.05	O24	−1.06

Table S6 Bond valence sum values for oxygen atoms in **InOC-41**.

In1	3.03	In2	2.99	In3	3.21
In4	3.03	In5	3.09	In6	3.03
In7	3.02	In8	3.08	In9	2.88
In10	2.94	In11	2.93	In12	2.95
In13	2.94	In14	2.93	In15	3.25
O2	−1.97	O3	−2.05	O4	−2.00
O6	−1.74	O8	−1.89	O9	−1.98
O10	−1.94	O11	−2.01	O15	−1.15
O20	−1.13	O26	−1.08		

Table S7 Bond valence sum values for oxygen atoms in **InOC-42**.

In1	3.05	In2	3.04	In3	3.10
In4	3.08	In5	2.96	In6	2.90
In7	2.84	In8	2.96	In9	3.13
In10	3.07	In11	2.69	In12	2.89
In13	2.94	In14	2.81	In15	3.19
O1	−1.88	O3	−1.98	O4	−2.08
O5	−2.14	O8	−1.75	O11	−1.90
O18	−1.98	O21	−2.02	O7	−1.08
O10	−1.10	O17	−1.11		

III. Additional structural pictures

(a) (b)

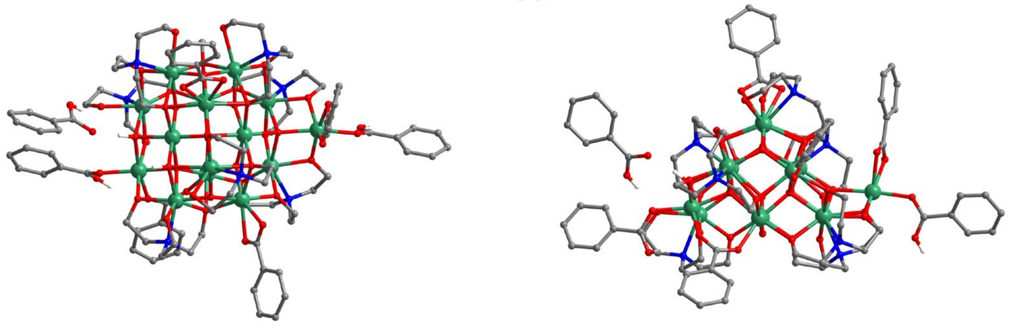

Figure S1 Molecular structure of **InOC-38** in top view **(a)** and side view **(b)**. Some H atoms are omitted for clarity.

(a) (b)

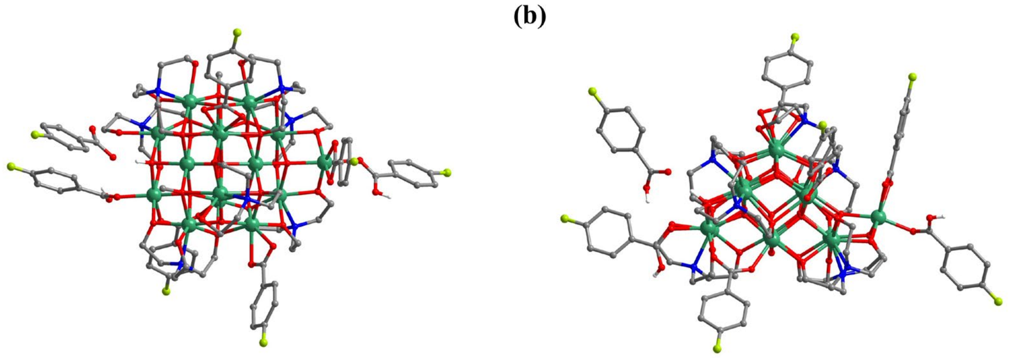

Figure S2 Molecular structure of **InOC-39** in top view **(a)** and side view **(b)**. Some H atoms are omitted for clarity.

(a) (b)

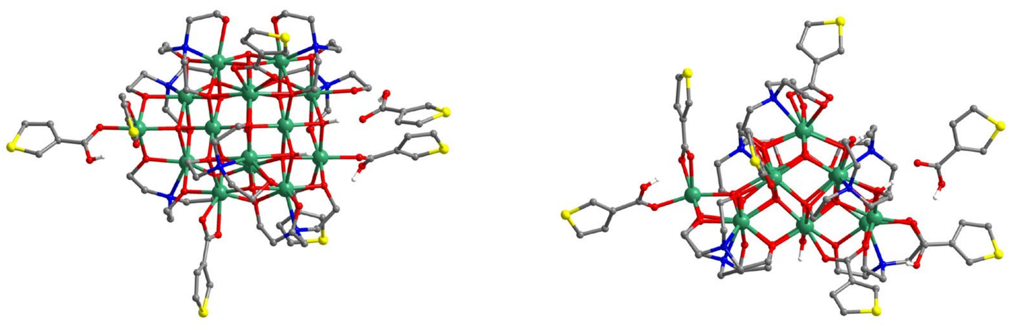

Figure S3 Molecular structure of **InOC-40** in top view **(a)** and side view **(b)**. Some H atoms are omitted for clarity.

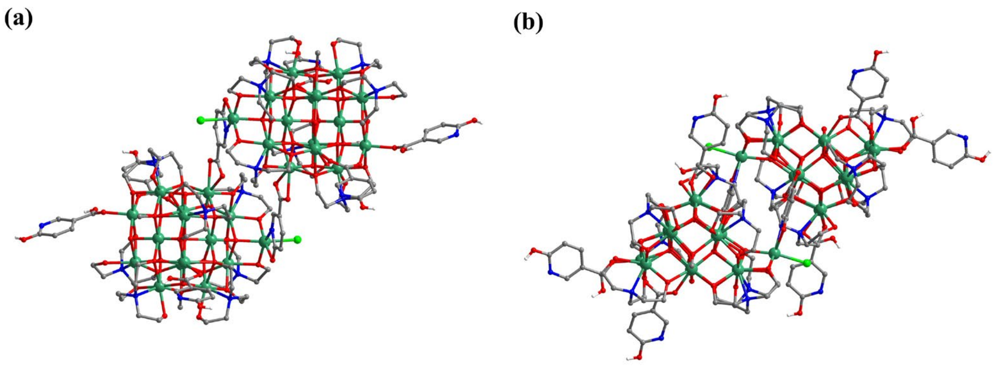

Figure S4 Molecular structure of **InOC-41** in top view **(a)** and side view **(b)**. Some H atoms are omitted for clarity.

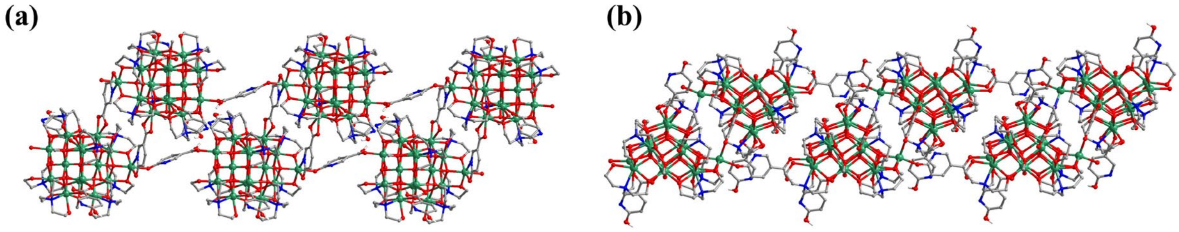

Figure S5 Molecular structure of **InOC-42** in top view **(a)** and side view **(b)**. Some H atoms are omitted for clarity.

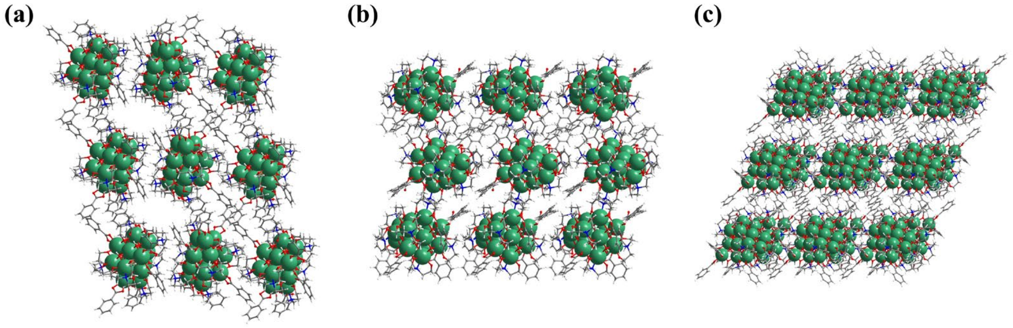

Figure S6 Packing mode of **InOC-38** along axis **a**, **b** and **c**.

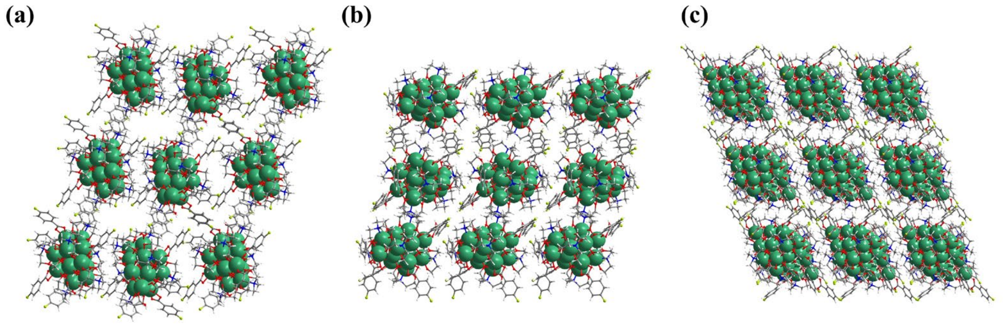

Figure S7 Packing mode of **InOC-39** along axis **a**, **b** and **c**.

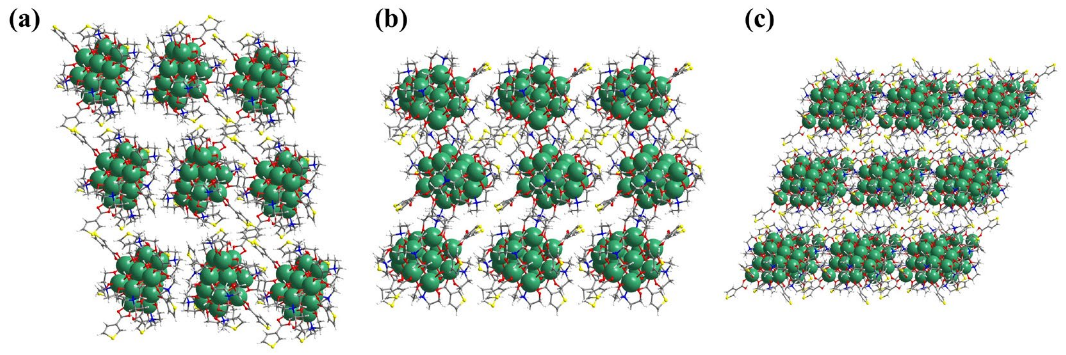

Figure S8 Packing mode of **InOC-40** along axis **a**, **b** and **c**.

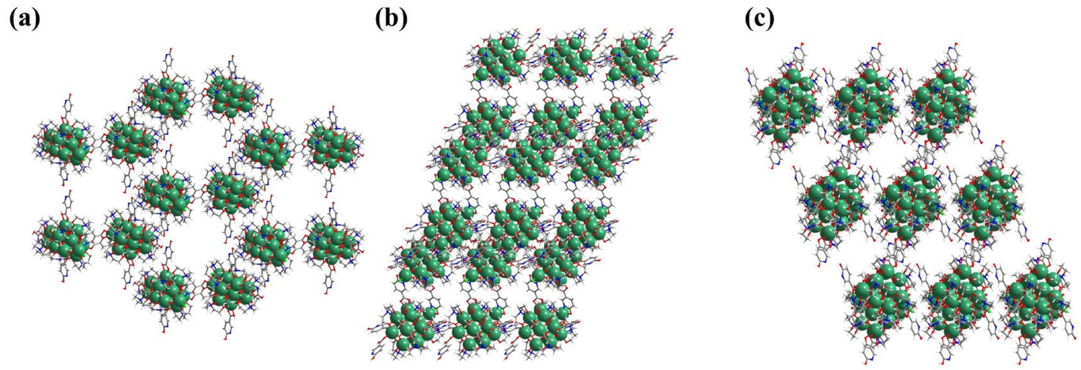

Figure S9 Packing mode of **InOC-41** along axis **a**, **b** and **c**.

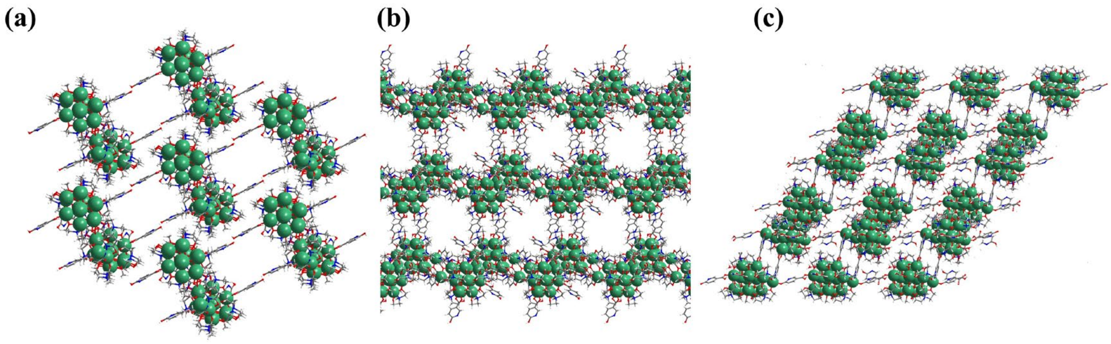

Figure S10 Packing mode of **InOC-42** along axis **a**, **b** and **c**.

IV. Supramolecular interaction analysis

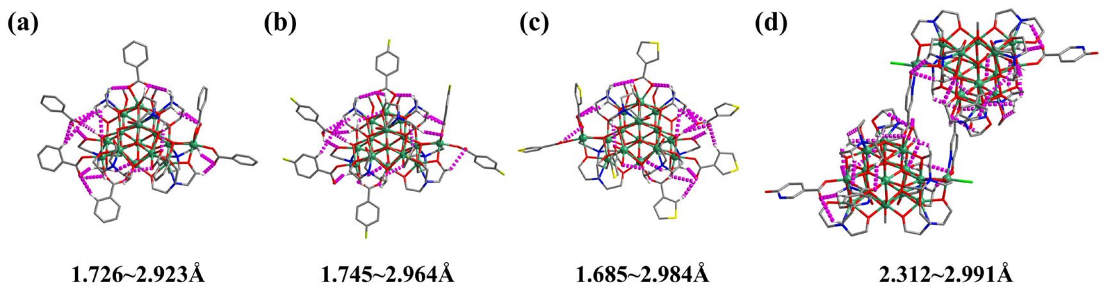

Figure S11 Intermolecular hydrogen bonds of **InOC-38** to **InOC-41**.

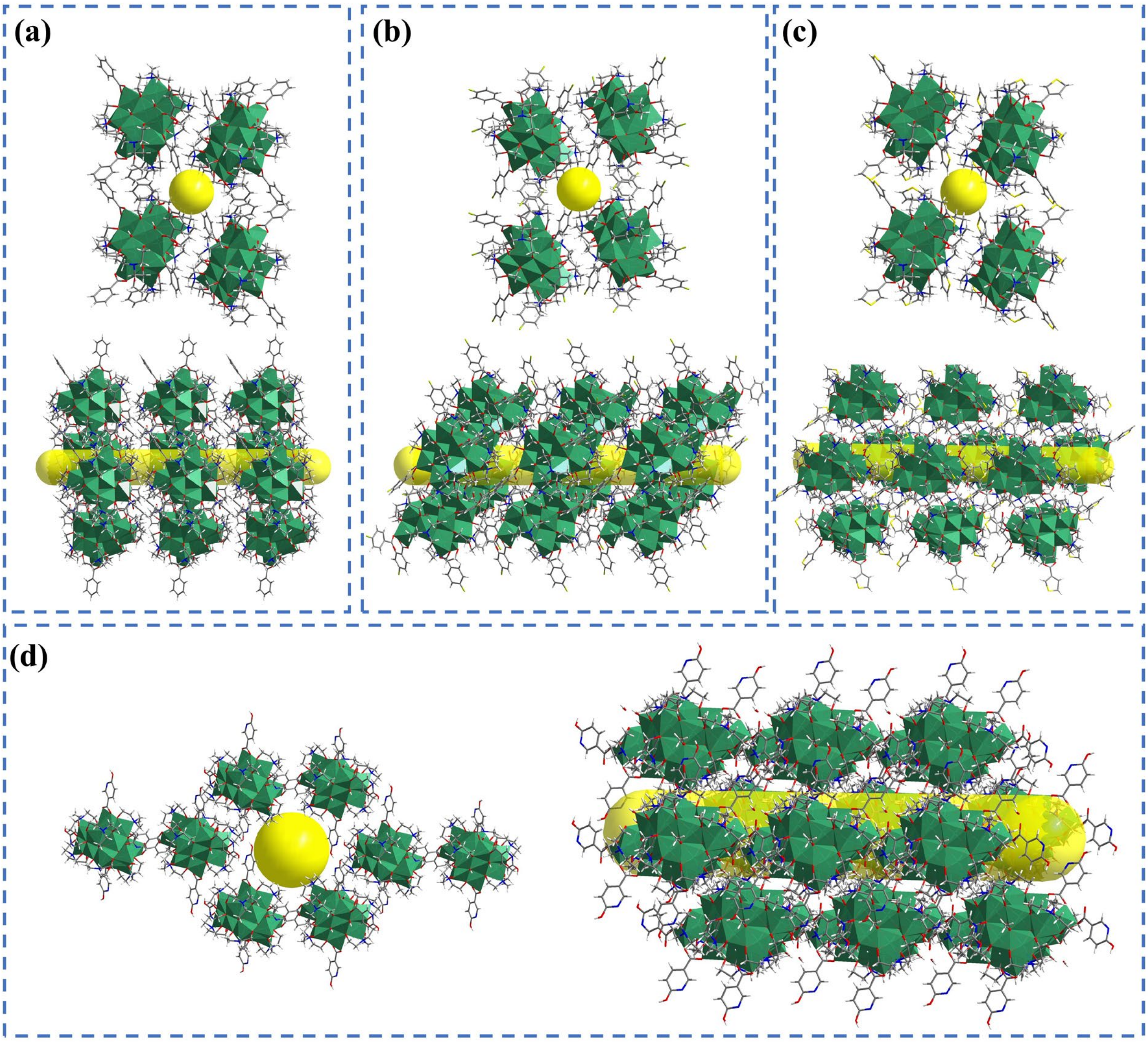

Figure S12 The packing channel diagram of **InOC-38** to **InOC-41** along axis **a**.

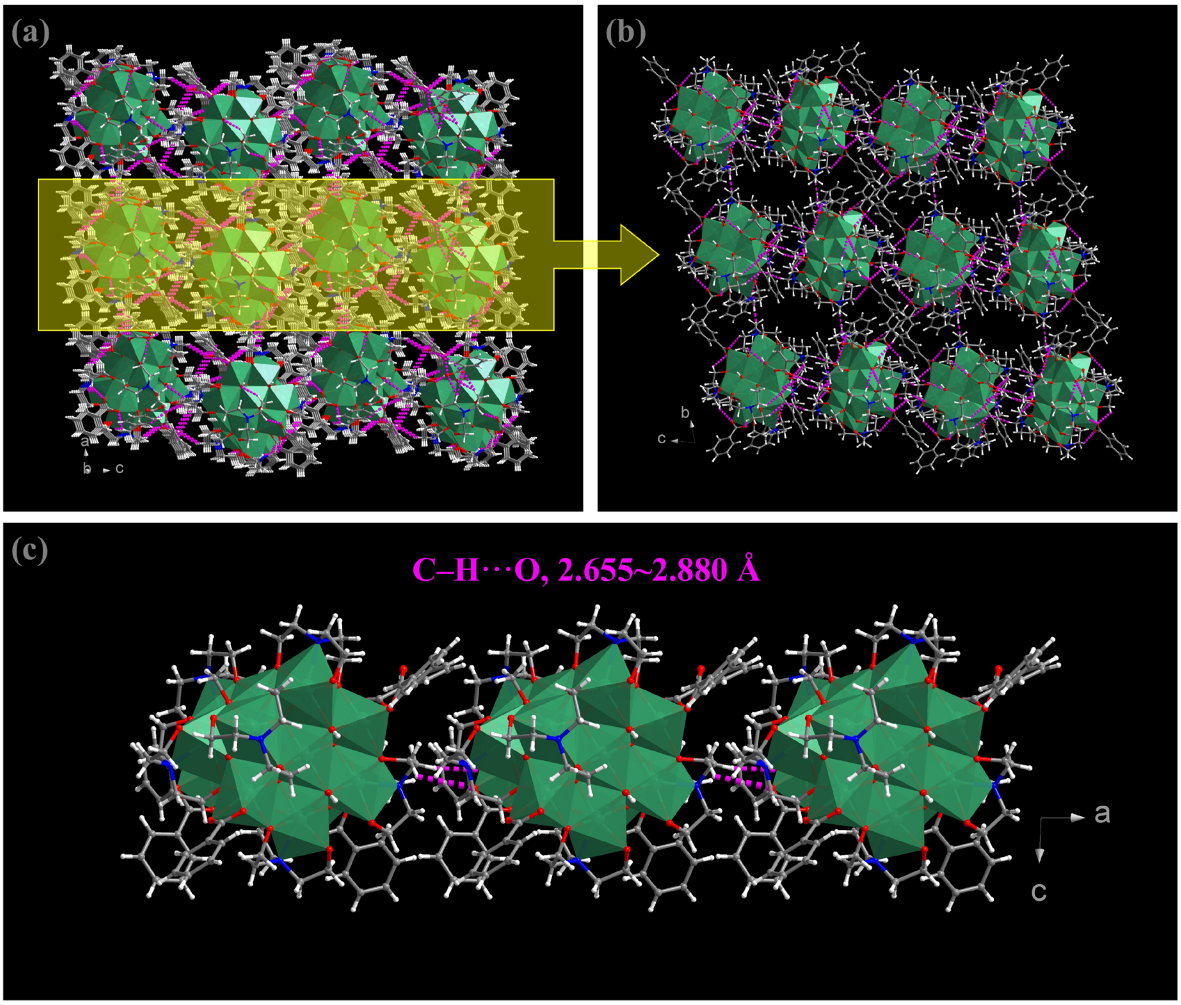

Figure S13 (a) Supramolecular interactions (hydrogen bond highlighted in dotted pink line); **(b)** Hydrogen bond interactions (including inter- and intra-cluster) in *bc* plane; **(c)** Intermolecular hydrogen bond interactions in **InOC-38.**

Due to the abundant hydrogen bond interactions (C-H ••• O, 2.370~3.889 Å), each cluster are connected into 2D layers in *bc* plane, furthermore, intermolecular hydrogen bond (C-H ••• O, 2.655~2.880 Å) facilitate them into 3D supramolecular structure.

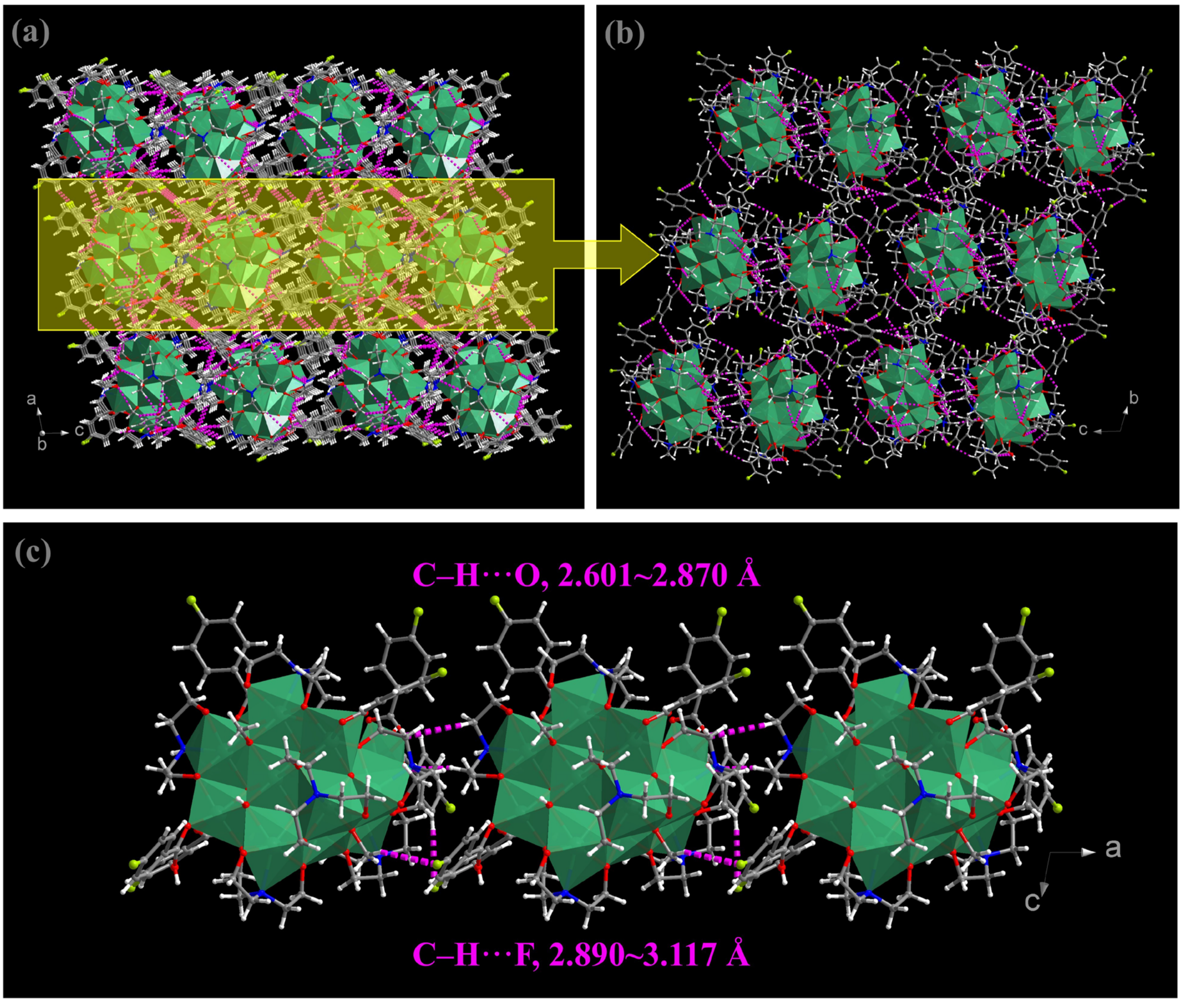

Figure S14 (a) Supramolecular interactions (hydrogen bond highlighted in dotted pink line); **(b)** Hydrogen bond interactions (including inter- and intra-cluster) in *bc* plane; **(c)** Intermolecular hydrogen bond interactions in **InOC-39.**

Due to the abundant hydrogen bond interactions (C-H ••• O, 2.504~2.978 Å and C-H ••• F, 2.297~2.890 Å), each cluster are connected into 2D layers in *bc* plane, furthermore, intermolecular hydrogen bond (C-H ••• O, 2.601~2.870 Å and C-H ••• F, 2.890~3.117 Å) facilitate them into 3D supramolecular structure.

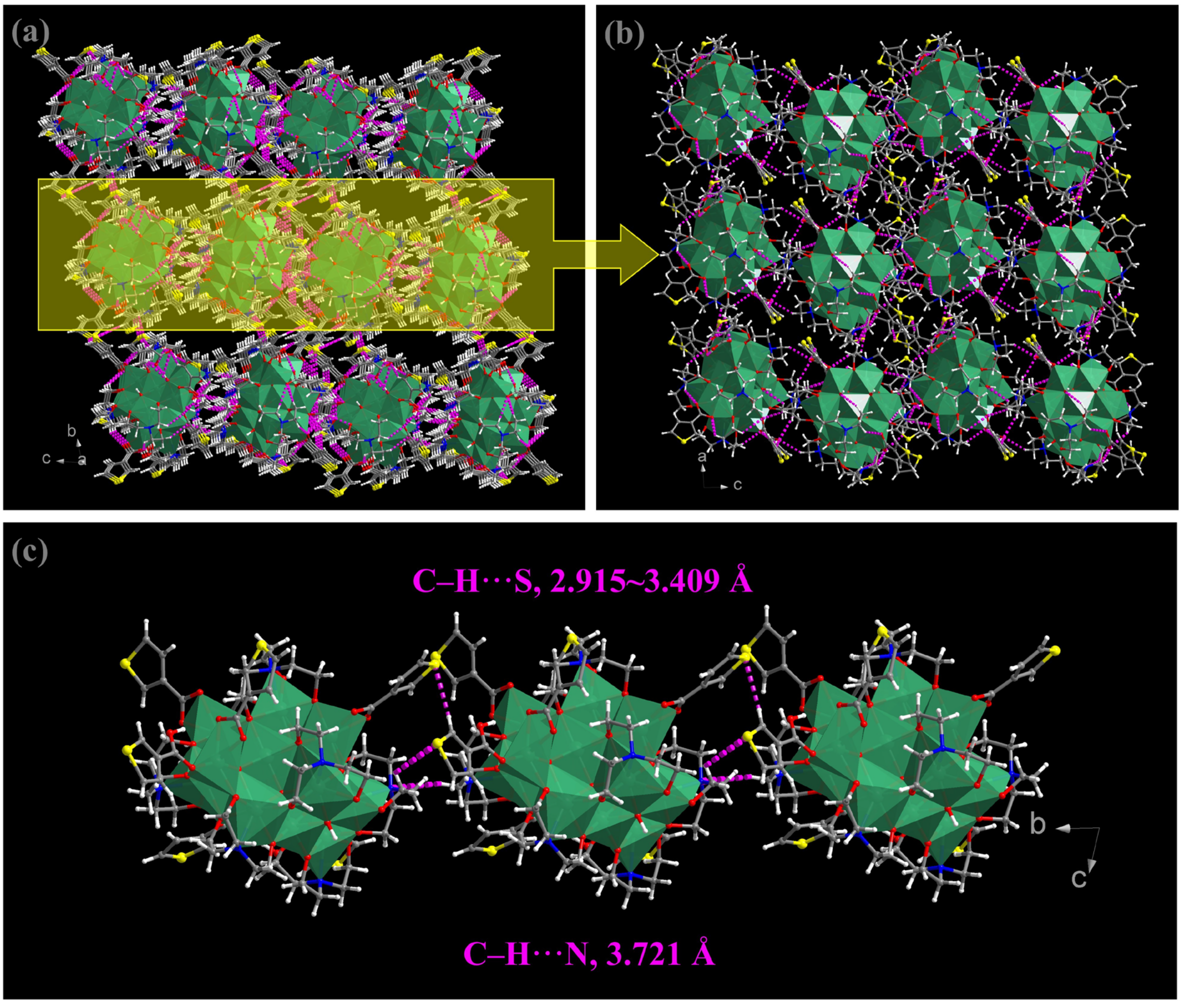

Figure S15 (a) Supramolecular interactions (hydrogen bond highlighted in dotted pink line); **(b)** Hydrogen bond interactions (including inter- and intra-cluster) in *ac* plane; **(c)** Intermolecular hydrogen bond interactions in **InOC-40.**

Due to the abundant hydrogen bond interactions (C-H ••• O, 2.328~2.939 Å and C-H ••• S, 2.806 Å), each cluster are connected into 2D layers in *ac* plane, furthermore, intermolecular hydrogen bond (C-H ••• S, 2.915~3.409 Å and C-H ••• N, 3.721 Å) facilitate them into 3D supramolecular structure.

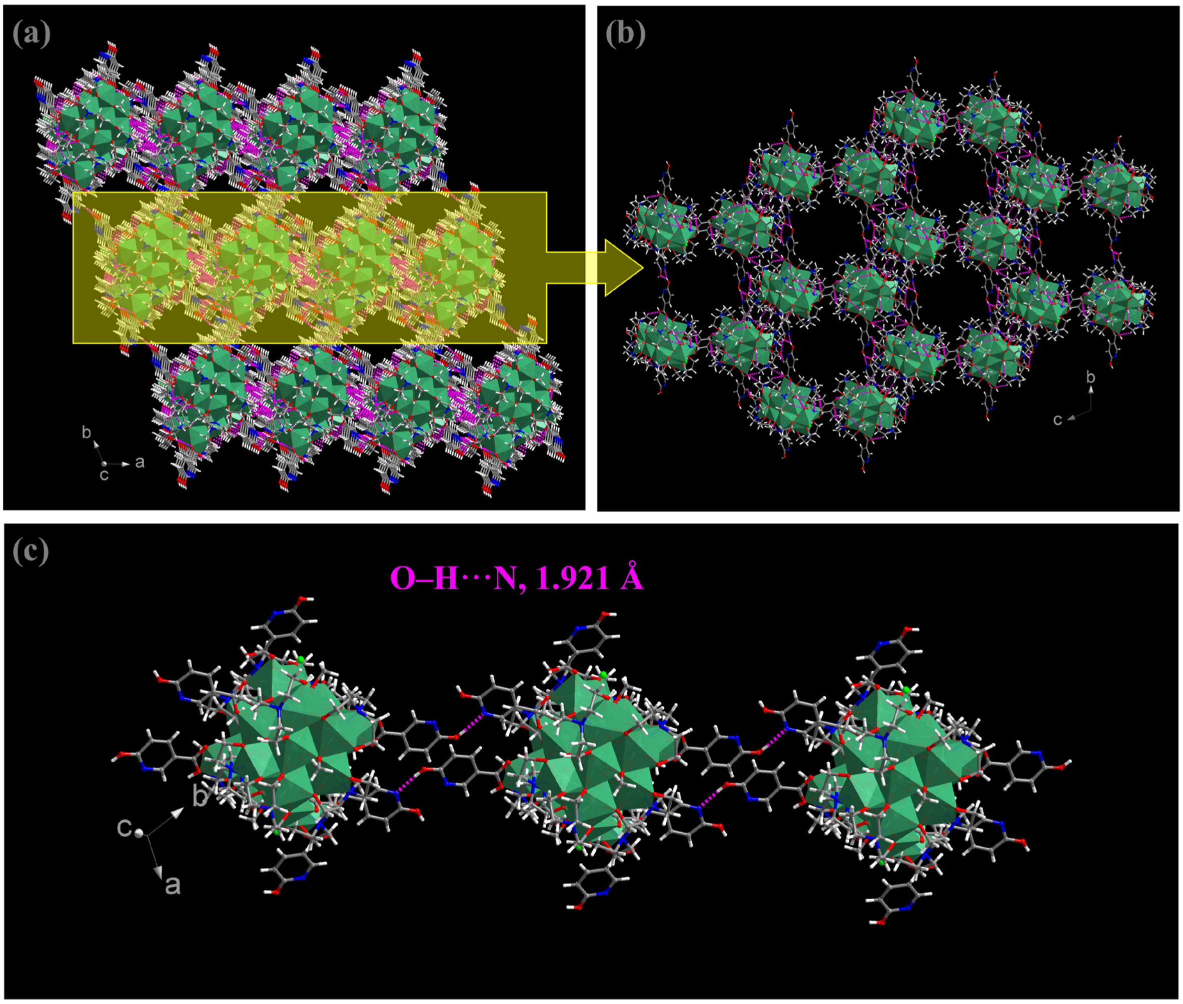

Figure S16 (a) Supramolecular interactions (hydrogen bond highlighted in dotted pink line); **(b)** Hydrogen bond interactions (including inter- and intra-cluster) in *bc* plane; **(c)** Intermolecular hydrogen bond interactions in **InOC-41.**

Due to the abundant hydrogen bond interactions (C-H ••• O, 2.303~2.935 Å), each cluster are connected into 2D layers in *bc* plane, furthermore, intermolecular hydrogen bond (O-H ••• N, 1.921 Å) facilitate them into 3D supramolecular structure.

V. Powder-XRD

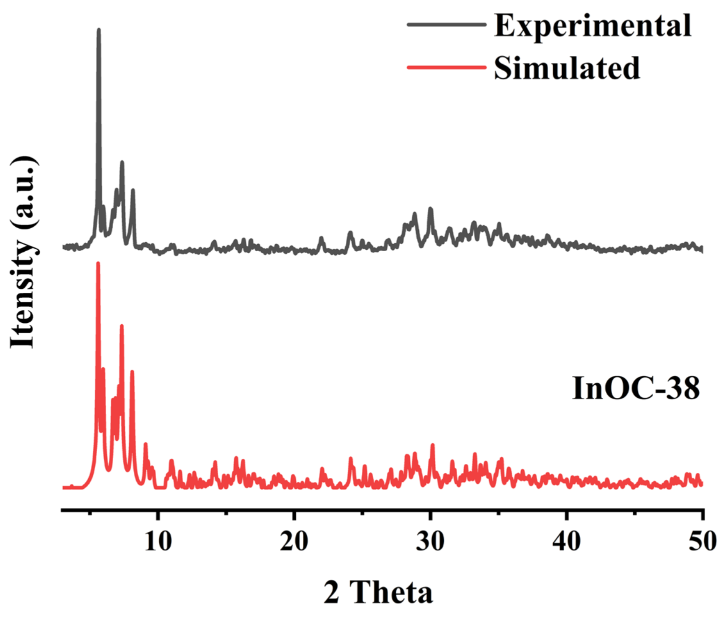

Figure S17 P-XRD analysis for **InOC-38**.

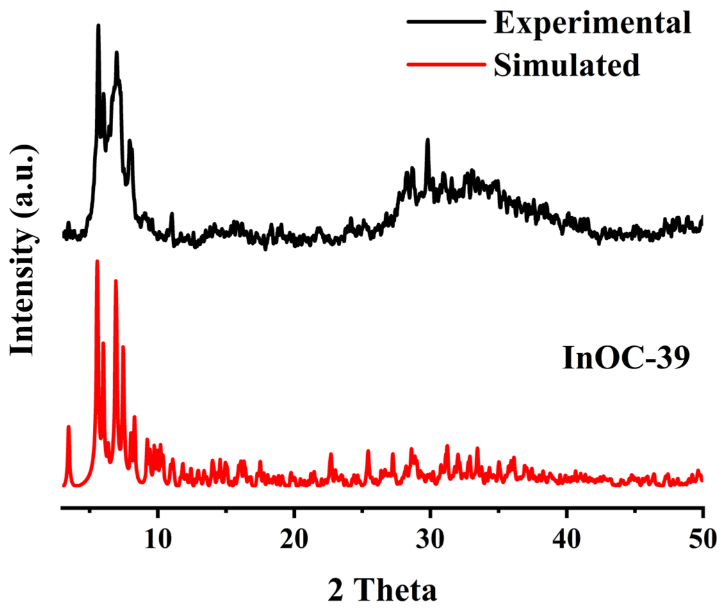

Figure S18 P-XRD analysis for **InOC-39**.

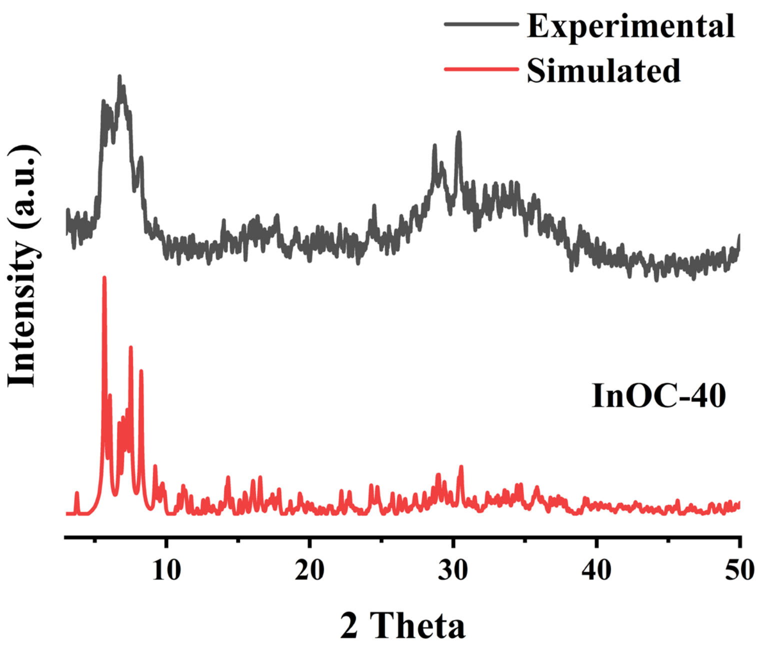

Figure S19 P-XRD analysis for **InOC-40**.

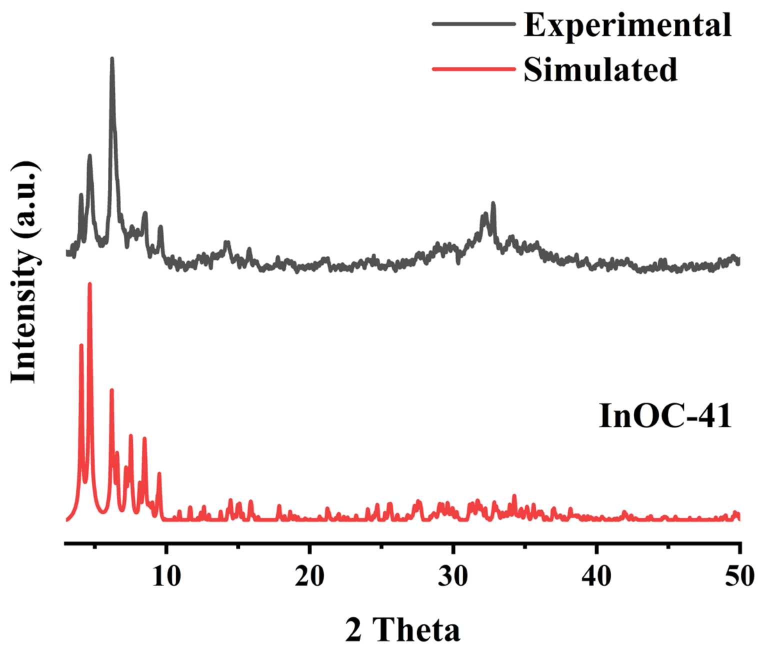

Figure S20 P-XRD analysis for **InOC-41**.

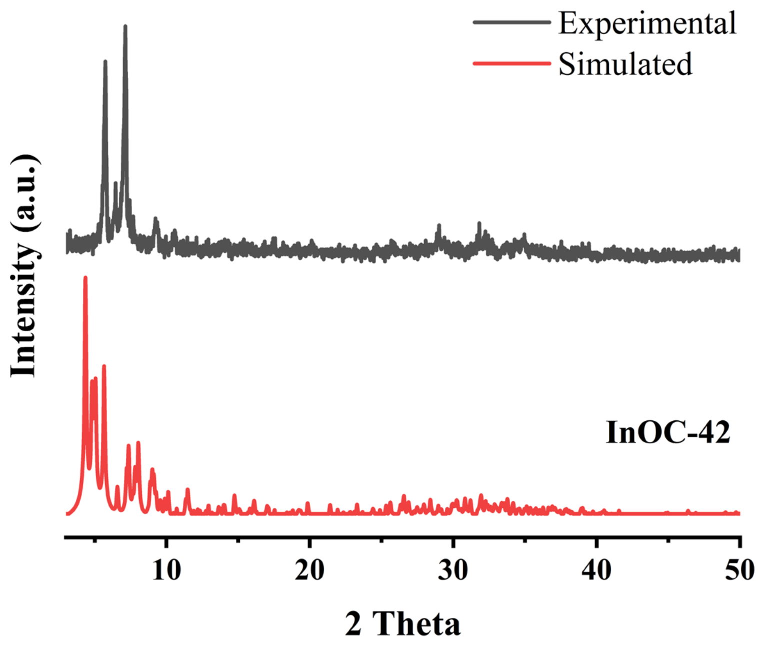

Figure S21 P-XRD analysis for **InOC-42**.

VI. The Energy Dispersive X-Ray Spectroscopy (EDS) spectra

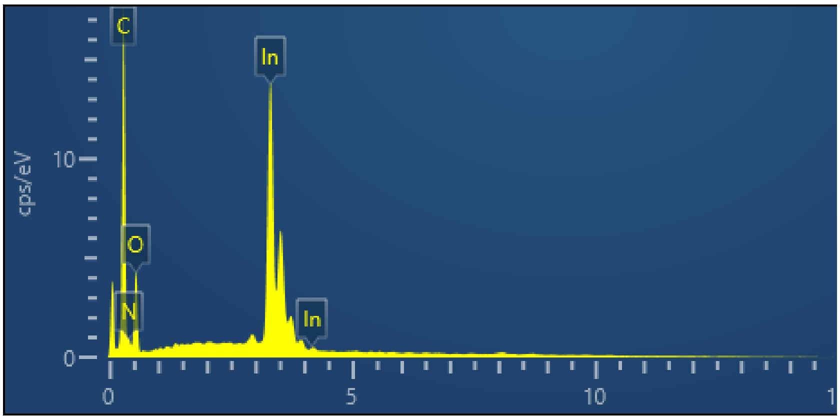

Figure S22 The EDS spectrum of **InOC-38**.

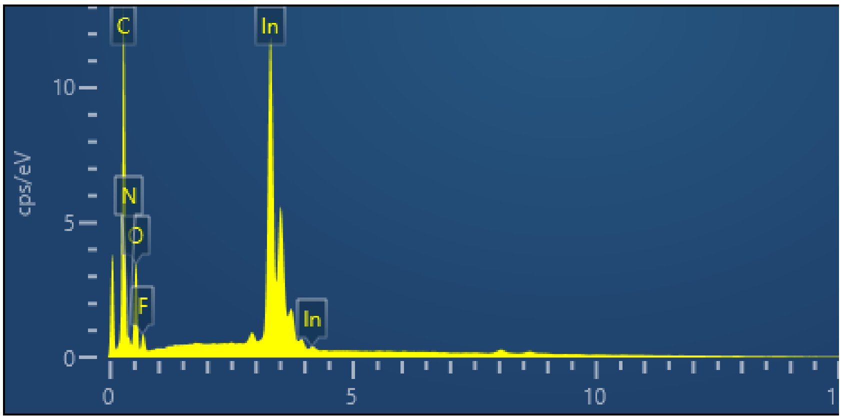

Figure S23 The EDS spectrum of **InOC-39**.

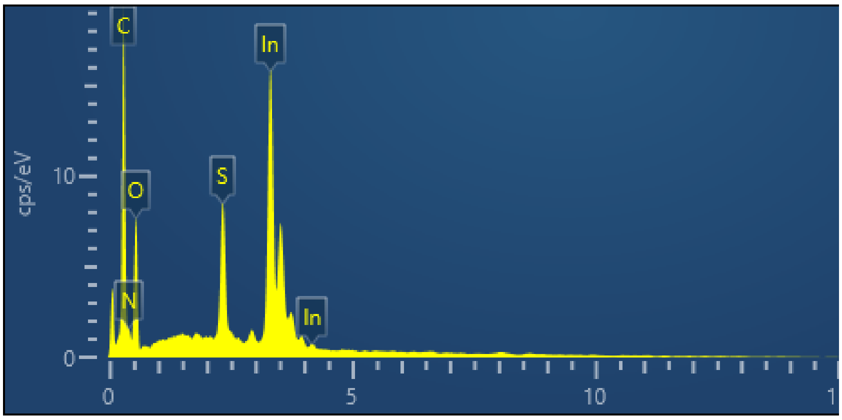

Figure S24 The EDS spectrum of **InOC-40**.

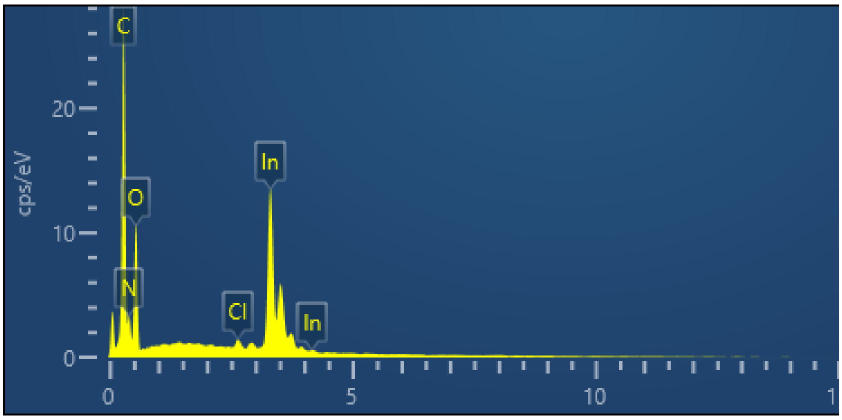

Figure S25 The EDS spectrum of **InOC-41**.

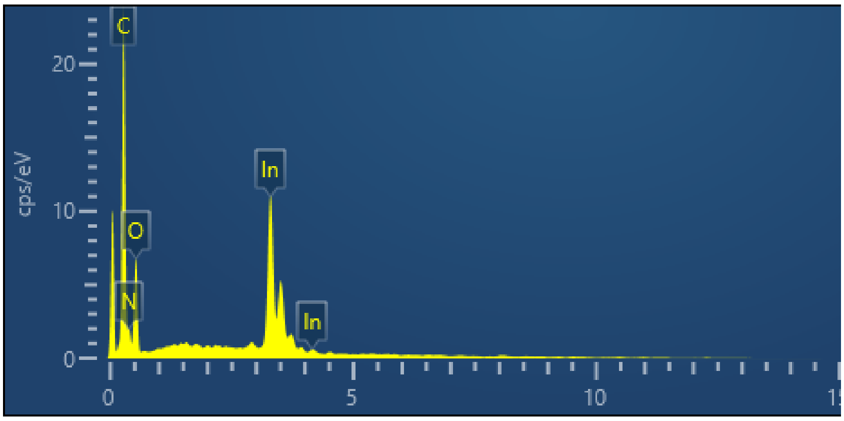

Figure S26 The EDS spectrum of **InOC-42**

VII. Thermogravimetrical analysis (TGA)

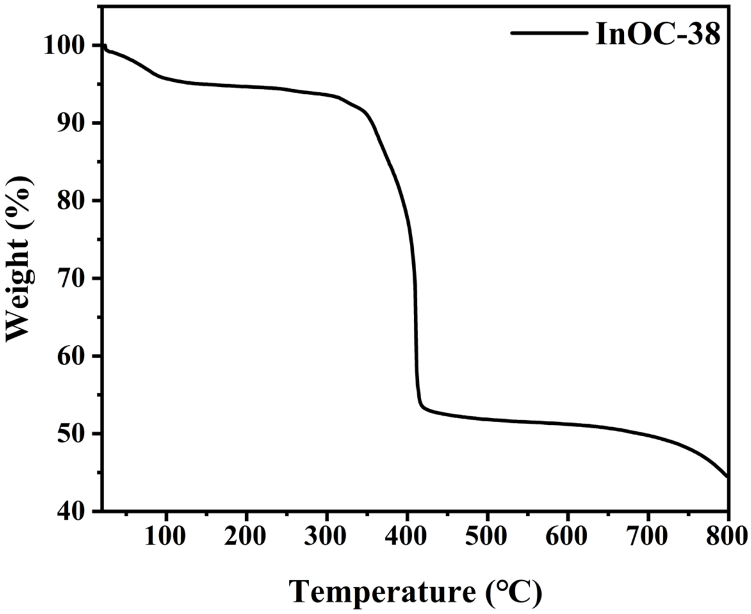

Figure S27 TG curve of compound **InOC-38** from room temperature to 800 °C under N_2 atmosphere.

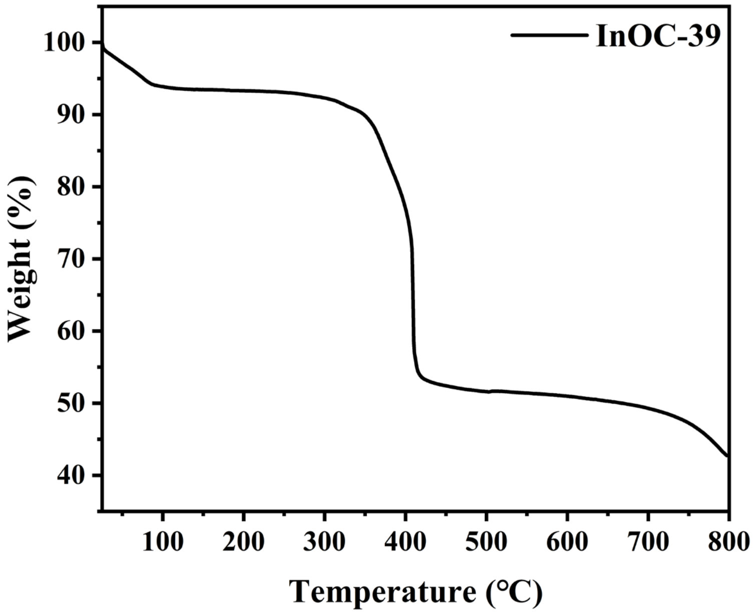

Figure S28 TG curve of compound **InOC-39** from room temperature to 800 °C under N_2

atmosphere.

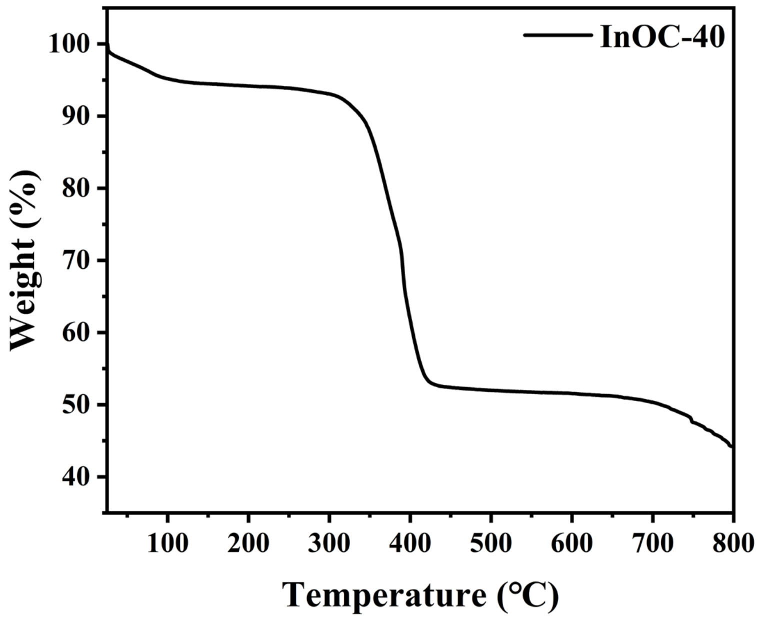

Figure S29 TG curve of compound **InOC-40** from room temperature to 800 °C under N_2 atmosphere.

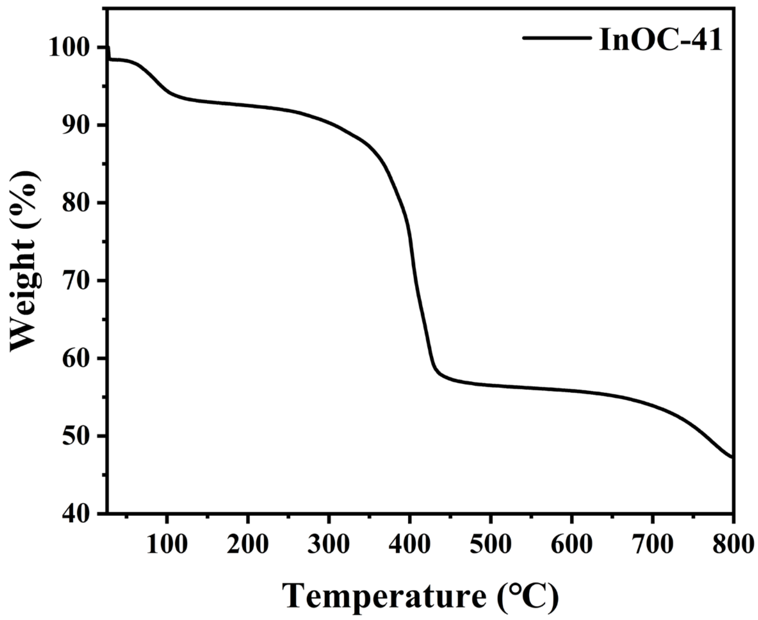

Figure S30 TG curve of compound **InOC-41** from room temperature to 800 °C under N_2 atmosphere.

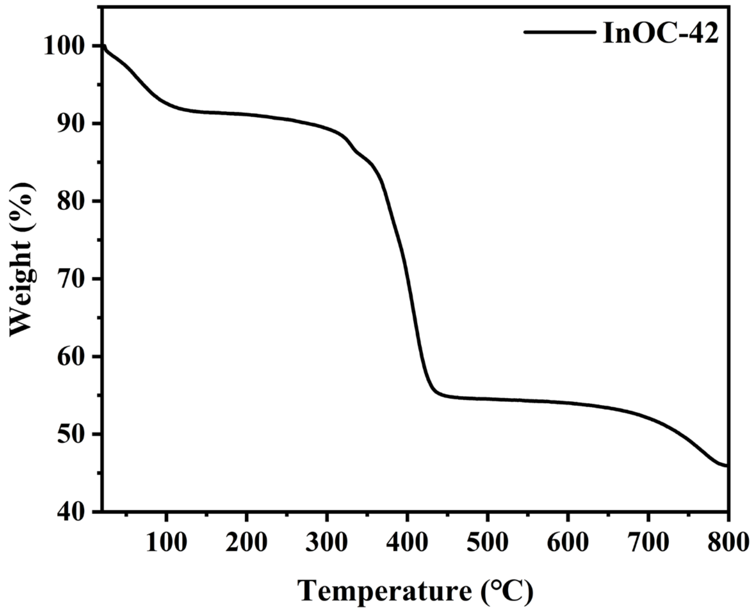

Figure S31 TG curve of compound **InOC-42** from room temperature to 800 °C under N_2 atmosphere.

VIII. Solid-state UV absorption spectra

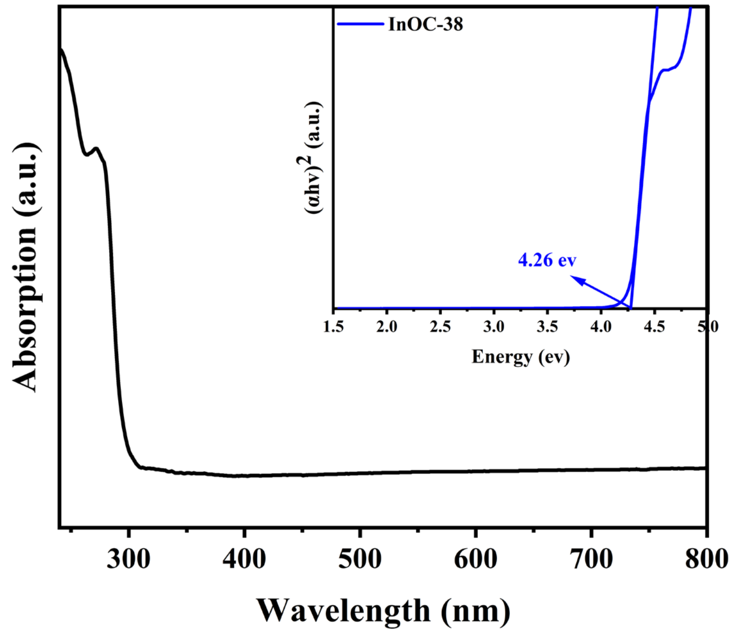

Figure S32 Solid-state UV absorption spectrum of **InOC-38.**

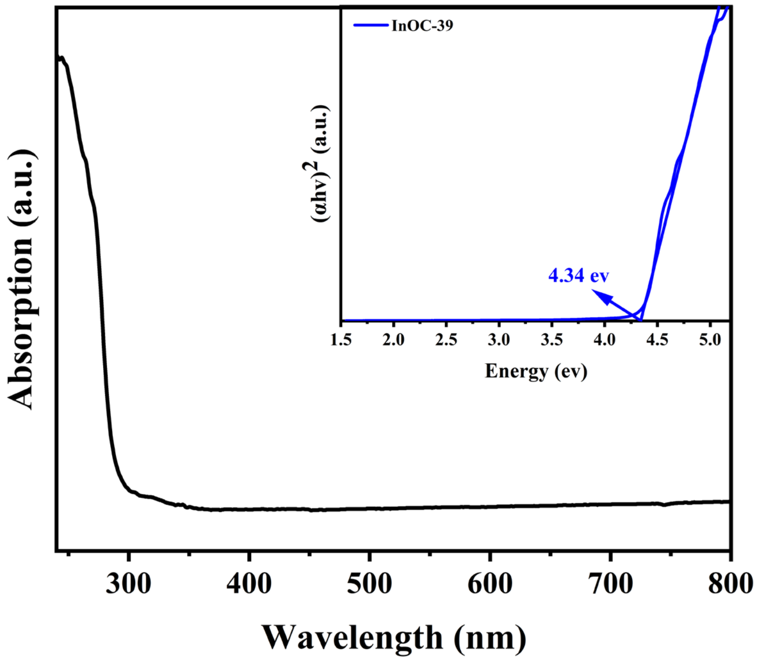

Figure S33 Solid-state UV absorption spectrum of **InOC-39.**

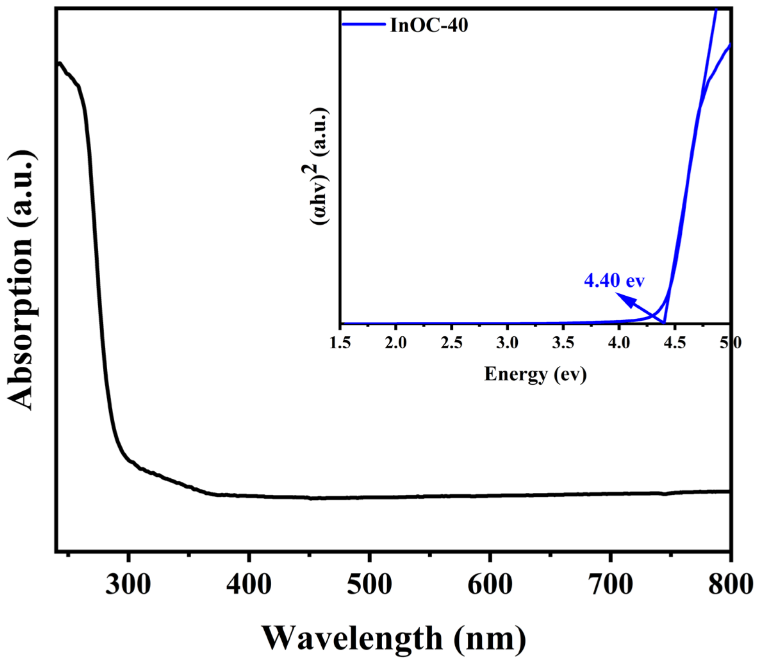

Figure S34 Solid-state UV absorption spectrum of **InOC-40.**

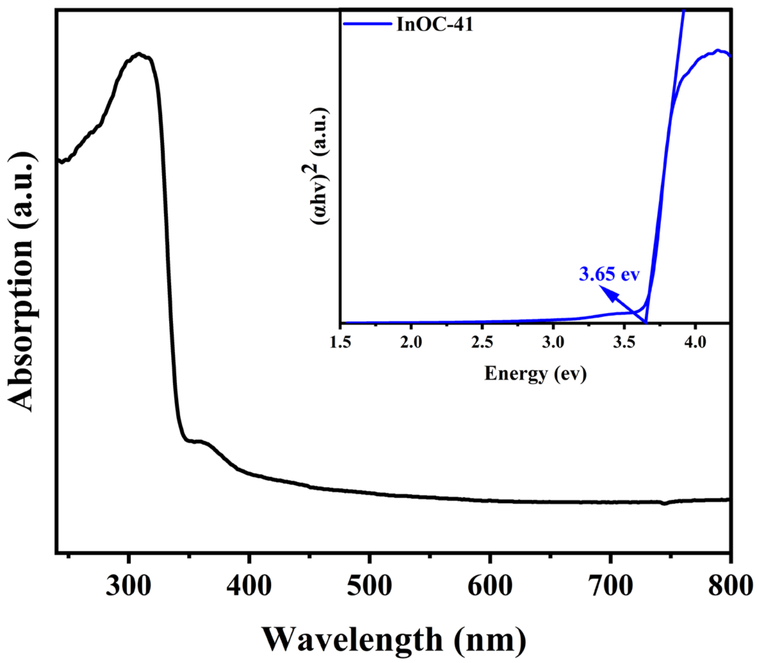

Figure S35 Solid-state UV absorption spectrum of **InOC-41.**

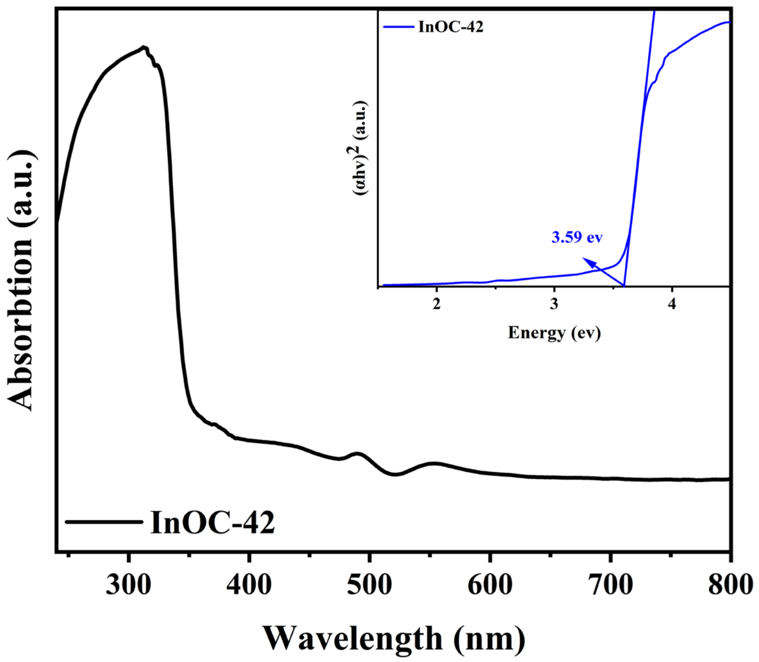

Figure S36 Solid-state UV absorption spectrum of **InOC-42.**

IX. IR spectra

The FT-IR spectra of **InOC-38** to **InOC-42** have been recorded between 4000 and 400 cm^{-1}. The spectra of these compounds exhibit the bands from 800-400 cm^{-1} characteristic for vibrations of M-O-M bonds (M: In). The aboard absorption band from 1540-1560 cm^{-1} stem from the $\nu_{as}(CO_2^-)$ stretching and the band from 1390-1410 cm^{-1} is assigned to the $\nu_s(CO_2^-)$ in **InOC-38** to **InOC-42 (Fig. S37-S41)**.The absorption peaks at 2950 cm^{-1} and 2860 cm^{-1} belong to C-H stretching vibration of benzene ring and C－H stretching vibration of alkanes, respectively in **InOC-38** to **InOC-42 (Fig. S37-S41)**. The vibration at 1230 cm^{-1} (in **InOC-39**)is assigned to the C－F vibration of the aromatic ring separately (**Fig. S38**).

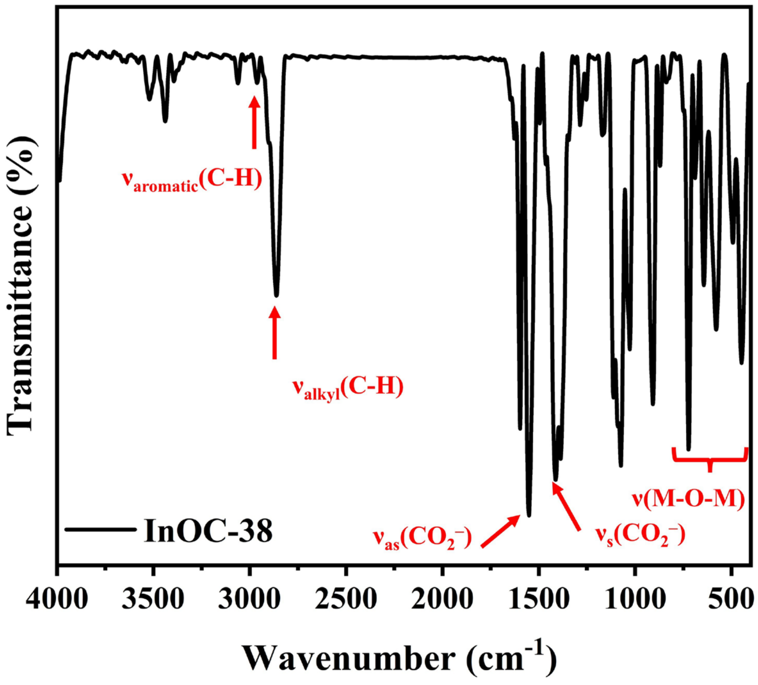

Figure S37 FT-IR spectrum of **InOC-38**.

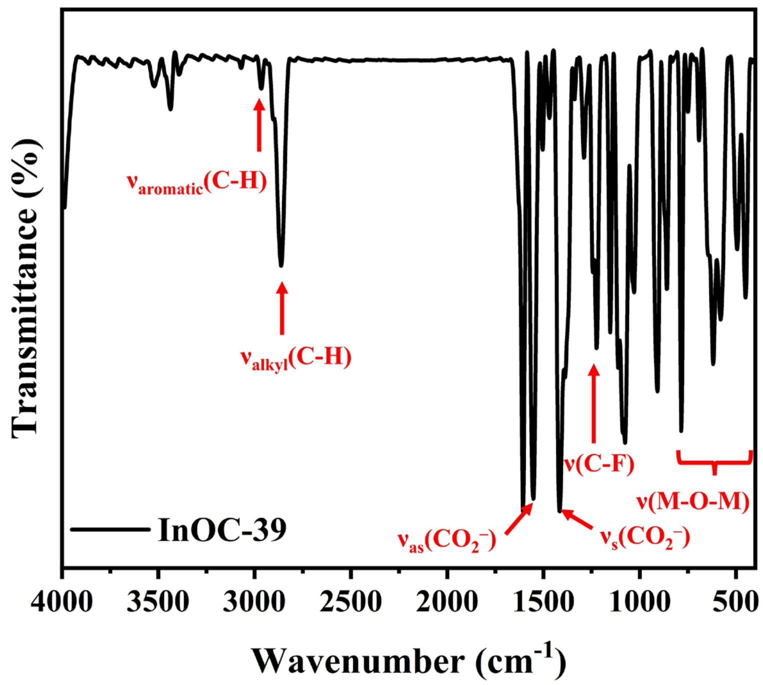

Figure S38 FT-IR spectrum of **InOC-39**.

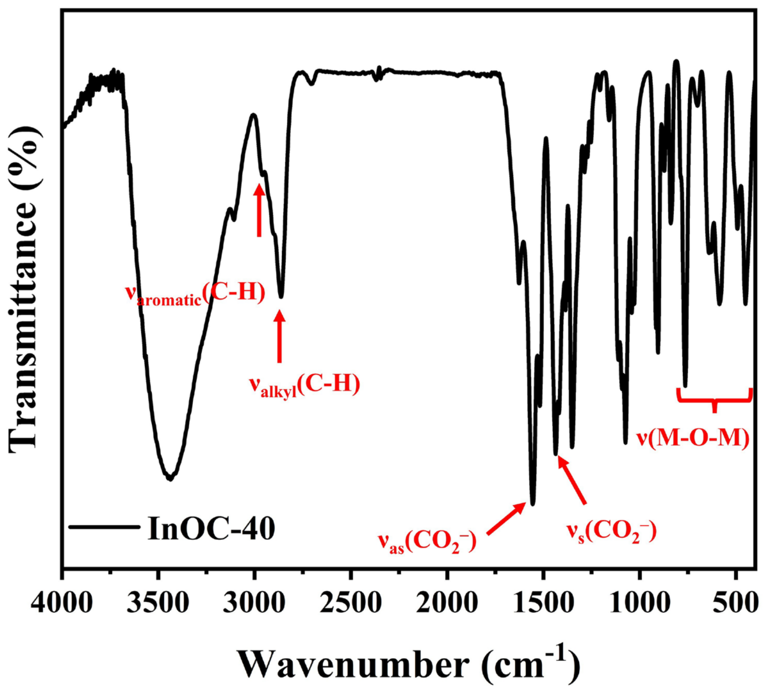

Figure S39 FT-IR spectrum of **InOC-40**.

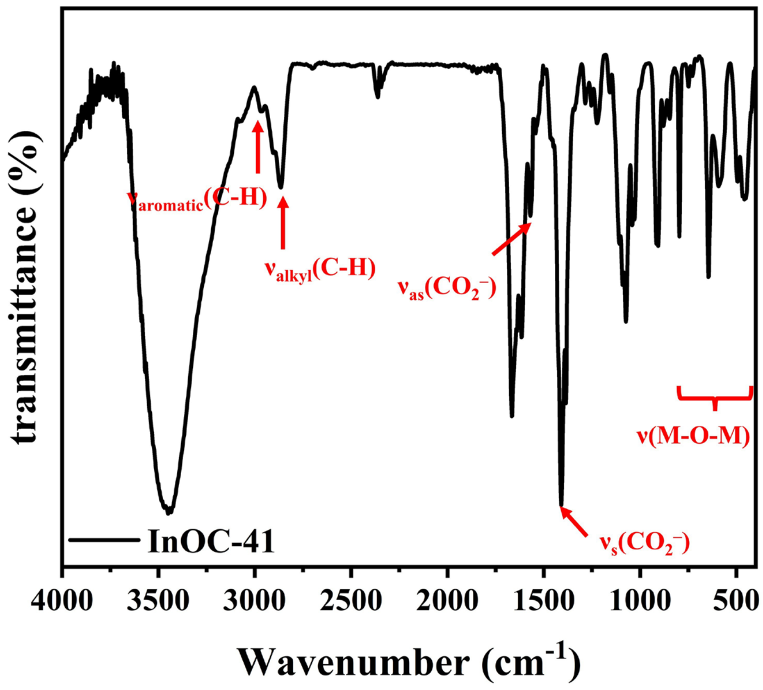

Figure S40 FT-IR spectrum of **InOC-41**.

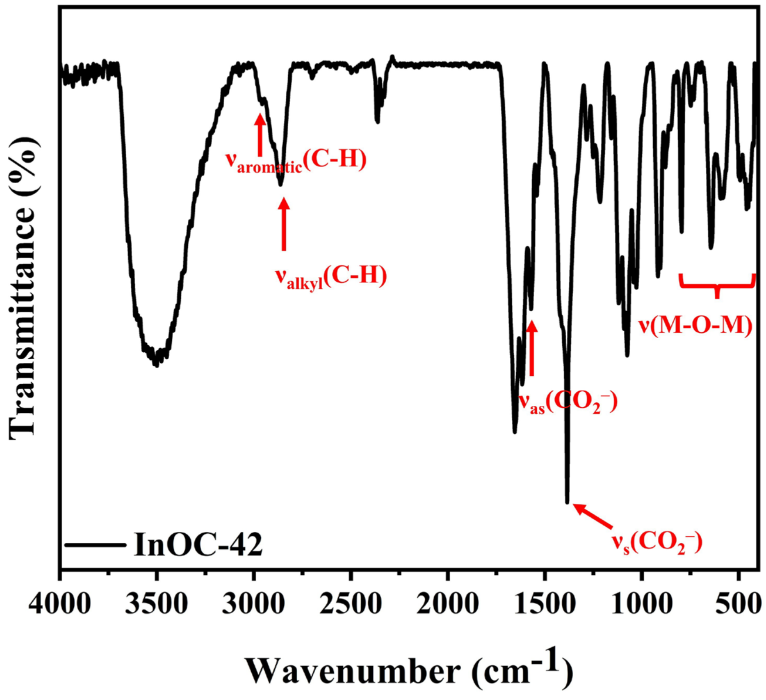

Figure S41 FT-IR spectrum of **InOC-42**.

X. The repeatability and stability of Z-scan measurements

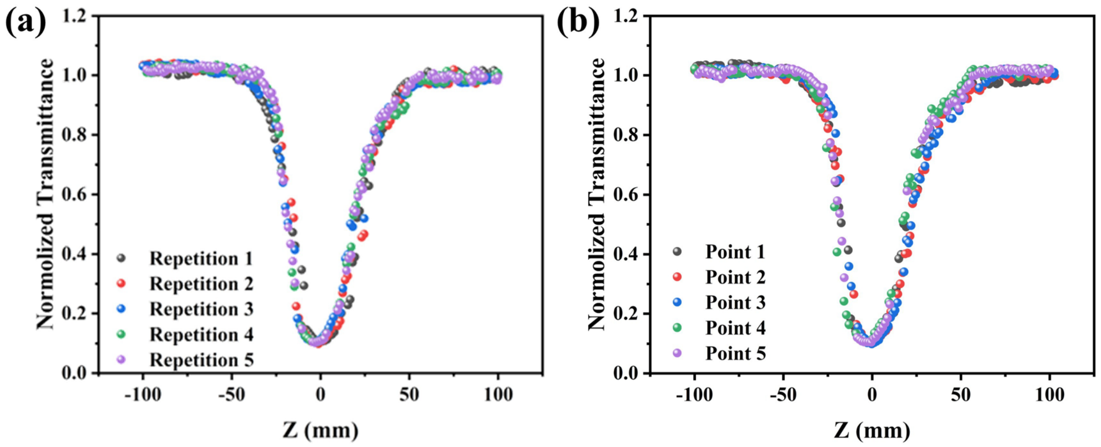

Figure S42 The repeatability of third-order NLO responses for **InOC-38** through 5 cycles (a) and 5 different points (b).

Such responses can be repeated and present consistent T_{min} values, showing that the prepared InOCs@PDMS films are featured with excellent chemical and physical stability.

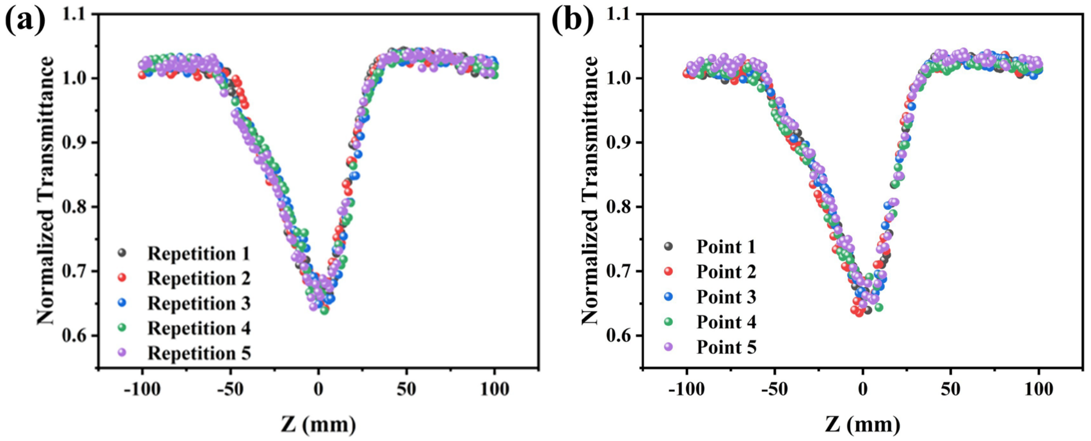

Figure S43 The repeatability of third-order NLO responses for **InOC-39** through 5 cycles (a) and 5 different points (b).

Such responses can be repeated and present consistent T_{min} values, showing that the prepared InOCs@PDMS films are featured with excellent chemical and physical stability.

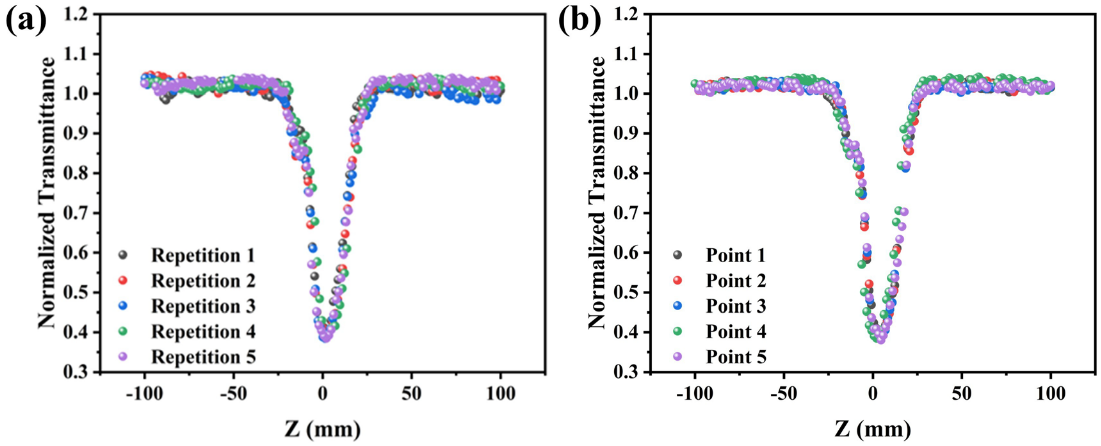

Figure S44 The repeatability of third-order NLO responses for **InOC-40** through 5 cycles (a) and 5 different points (b).

Such responses can be repeated and present consistent T_{min} values, showing that the prepared InOCs@PDMS films are featured with excellent chemical and physical stability.

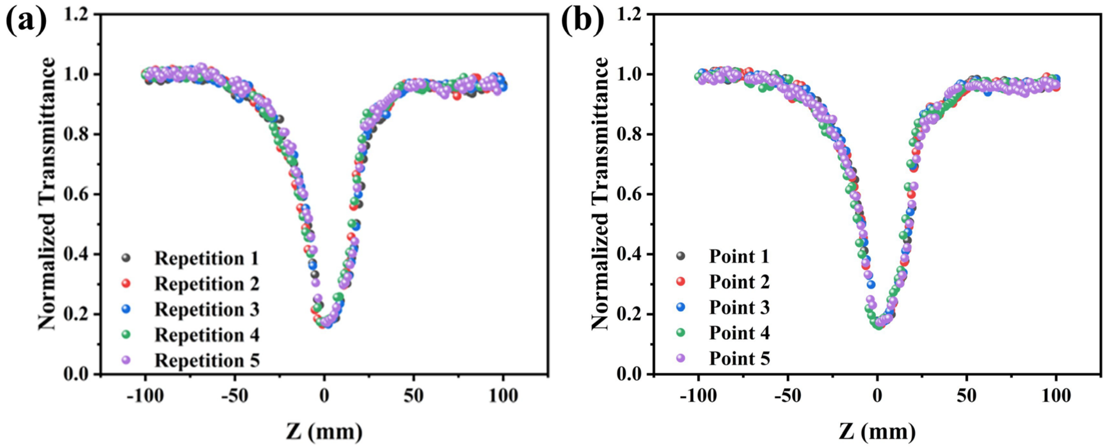

Figure S45 The repeatability of third-order NLO responses for **InOC-41** through 5 cycles (a) and 5 different points (b).

Such responses can be repeated and present consistent T_{min} values, showing that the prepared InOCs@PDMS films are featured with excellent chemical and physical stability.

Table S8 Comparison of third-order NLO properties with other clusters.

Samples	T_{min}	β(m/W)	F_{OL} (J/cm^2)	Compound color	Reference
**InOC-38**@PDMS $In_{15}(BA)_4(HBA)_2(C_6H_{12}O_3N)_7(\mu_4\text{-}O)_6(\mu_3\text{-}O)_2(\mu_3\text{-}OH)_2(\mu_2\text{-}OH)(\mu_3\text{-}OCH_3)$	0.11	$6.8*10^{-9}$	0.275	colorless	This work
**InOC-41**@PDMS $In_{30}(NA)_2(HNA)_2(H_2NA)_4(C_6H_{12}O_3N)_{14}(\mu_4\text{-}O)_{12}(\mu_3\text{-}O)_4(\mu_3\text{-}OH)_2(\mu_2\text{-}OH)_2(\mu_3\text{-}OCH_3)_4Cl_2$	0.17	$6.1*10^{-9}$	0.408	colorless	
**InOC-19@**PDMS $In_{10}Ni_8(\mu 3\text{-}OH)_{22}(NA)_2(CH_3CN)_4(H_2O)_2Cl_{28}$	0.21	$9.6*10^{-9}$	1.13	light green	1
**InOC-18@**PDMS $In_{10}Ni_8(\mu 3\text{-}OH)_{22}(BA)_2(CH_3CN)_4(H_2O)_2Cl_{28}$	0.43	$2.8*10^{-9}$	1.02	light green	
**PTC-373**@PDMS $[Zn(H_2O)_4]\cdot\{Zn_3(Zr_4L_6)[Zn_4(Bim)_4(im)_3(H_2O)](H_2O)_6\}\cdot(NO_3)$	0.10	$5.77*10^{-9}$	0.13	pale yellow	2
**PTC-349**@PDMS $[Ag_6Ti_{12}(PA)_{12}(BA)_6(\mu_2\text{-}O)_{12}(\mu_3\text{-}O)_6]$	0.5	$0.6*10^{-9}$	2.57	colorless	3
**PTC-350**@PDMS $[Ag_8Ti_{12}(PA)_{12}(\mu_2\text{-}O)12(\mu_3\text{-}O)_6(AC)_6(C_8H_4Cl)_2(H_2O)_2]$	0.43	$0.95*10^{-9}$	0.67	colorless	
**AlOC-93**@PDMS $[Al_{20}(phenol)_{20}(\mu_2\text{-}OH)_{10}(PyrC)_{30}]\cdot 6phenol$	0.58	$0.72*10^{-9}$	0.1154	pale yellow	4
**AlOC-95**@PDMS $[Al_{20}(phenol)_{20}(\mu_2\text{-}OH)_{10}(HPyrC)_{28}(MA)_2]\cdot 4phenol$	0.68	$0.48*10^{-9}$	0.498	pale yellow	
**ZnTPyP-1**@PDMS $[Zn_2(TPyP)(AC)_2]$	0.17	$13.50*10^{-9}$	0.79	Purple	5
**PTC-272**@KBr $Ti_6(\mu_2\text{-}O)_2(\mu_3\text{-}O)_2(PA)_8(Cat)_4\cdot 2HPA$	0.24	$9*10^{-8}$	0.669	black	6
**PTC-273**@KBr $Ti_{14}(\mu_2\text{-}O)_6(\mu_3\text{-}O)_6(PA)_{10}(Cat)_{10}(HCat)_2$	0.17	$11.7*10^{-8}$	0.157	black	

**Ag_{14}@Ti_{16}**@KBr $[Ag_{14}Ti_{16}O_{24}(FBC)_{28}(^{t}BUC{\equiv}C)_2](H_2O)_4(Toluene)_3$	0.41	-	0.815	light green	7
**$AgSn_{12}$**@KBr $Ag_2Sn_{24}O_{72}C_{132}N_{11}H_{284}$	0.29	$2.55*10^{-8}$	0.502	colorless	8
**C60**@PMMA	0.39	$1.25*10^{-8}$	3.13	purple-red	9
**$LaPc(CHO)_4$**@PMMA $C_{60}H_{32}ClN_8O_8La$	0.52	$4.5*10^{-9}$	5.26	dark green	
**LaPc-4(C_{60})**@PMMA $C_{308}H_{60}O_4N_{12}ClLa$	0.14	$2.9*10^{-8}$	2.34	brownish yellow	
**TOC-26**@PMMA $(nBuSn)_2(TEOA)_2(3\text{-}TPC)_2$	0.95	-	-	colorless	10
**TOC-27**@PMMA $(nBuSn)_2(TEOA)_2(BC)_2$	0.92	-	-	colorless	
**TOC-28**@PMMA $(nBuSn)_2(TEOA)_2(4HB_5CA)_2$	0.90	-	-	colorless	
**Zn-THPP**@DMF $Zn(N_2OC_{11}H_8)_4$	0.52	$4.6*10^{-6}$	-	colorless	11
**Pt-Ni cluster**@rGO	0.24	$1.98*10^{-11}$	1.42	light black	12
**CTGU-SnC-9@**KBr $[(n\text{-}BuSn)_{12}(OH)_8O_{12}\text{-}(APZA)_3]\cdot(APZA)\cdot DMF\cdot 5H_2O$	0.278	$3.3*10^{-8}$	0.492	colorless	13
**BOC-6@**PDMS $[Cu(II)_2B_{10}O_{12}(Qi)_8(L_4)_2]\cdot[CF_3SO_3]_2$ L=1,2-bis(4-pyridyl) ethane	0.23	$1.695*10^{-9}$	0.42	light yellow	14
**CTGU-SnC-16**@ $[(n\text{-}BuSn)_{12}Sn_6O_{20}(PZCl)_{16}F_4]\cdot 4H_2O$	0.57	$10.5*10^{-12}$	>0.19	colorless	15
**InOC-26@**PDMS $[Ti_{10}In_4Ag_6(SA)_{20}(SO_4)_4(DPPB)_3(CH_3CN)_4]$	0.41	$1.09*10^{-9}$	1.70	reddish - orange	16
**BOC-13@**PDMS $[Zr_2(Dmg)_4(CH_3OB_4O_5)_2BA_2]$	0.20	$5.7*10^{-9}$	0.624	colorless	17
**BOC-17**@PDMS $[Zr_2(Dmg)_4(4\text{-}FPh\text{-}B_3O_5)_2(Me\text{-}Pz)_2BA_2]$	0.32	$2.5*10^{-9}$	0.291	colorless	

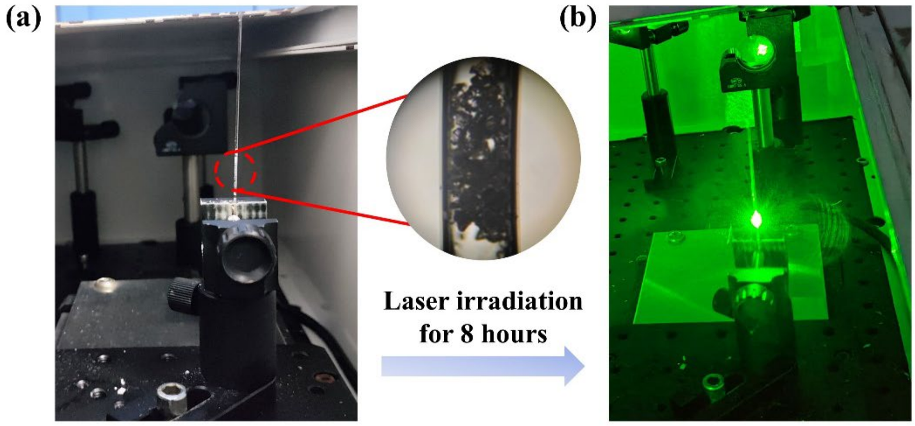

Figure S46 (a, b) The crystals **InOC-38, InOC-39, InOC-40** and **InOC-41** are placed in PDMS solution (without solidifying) with laser irradiation for 8 hours.

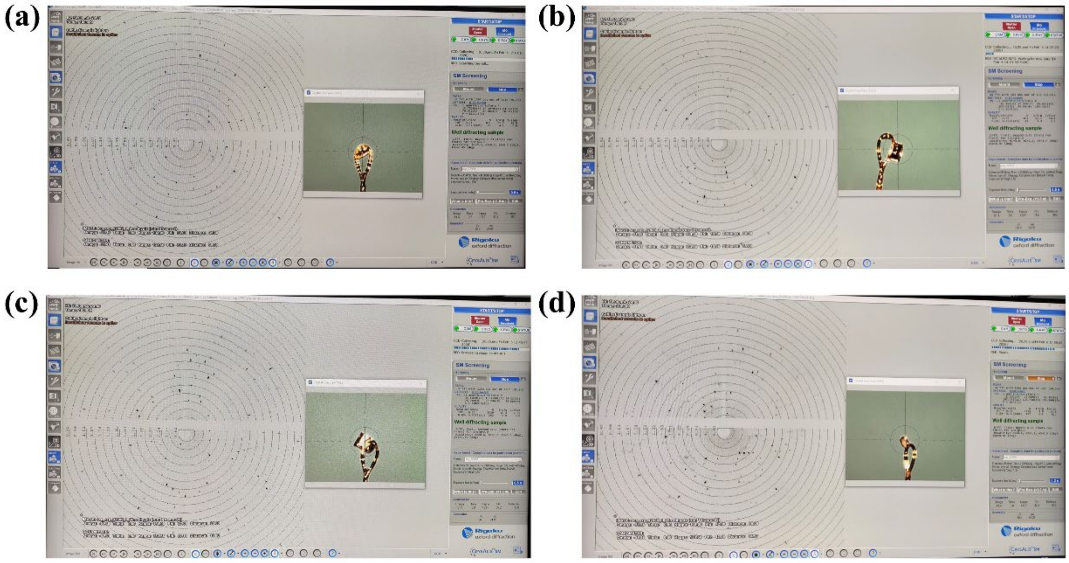

Figure S47 (a-d) **InOC-38-laser** to **InOC-41-laser** were characterized using S-XRD. The results demonstrate that their configurations remain the same after longtime laser radiation for 8 hours, which manifest their good stability and feasibility for real-world applications. (InOC-38 to InOC-41 after laser irradiation are abbreviated as InOC-38-laser to InOC-41-laser).

XI. Long-term stability testing.

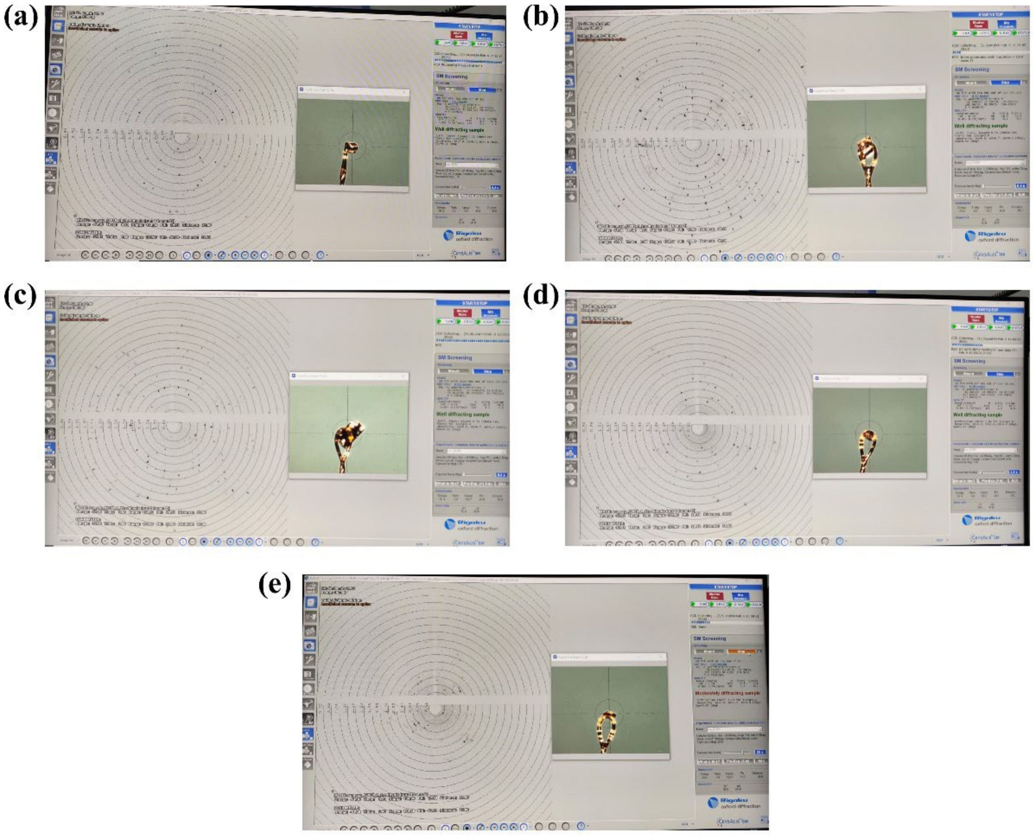

Figure S48 (a-e) The S-XRD images of **InOC-38 to InOC-42** crystal after half a year of storage.

XII. Uniformity of InOCs@PDMS film.

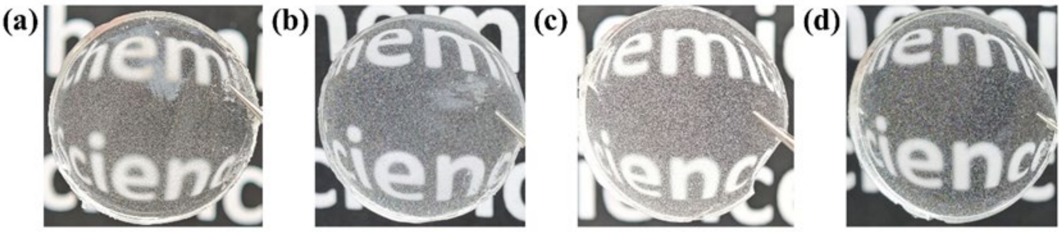

Figure S49 Photographs of PDMS substrate and InOCs@PDMS films under natural light.

Reference

1. X. Sun, X. Yi, J. Zhang and L. Zhang, *Chem. Mater.*, 2023, **35**, 5845-5853.
2. Y. F. Li, Y. P. He, Q. H. Li and J. Zhang, *Angew. Chem. Int. Ed.*, 2024, **63**, e202318806.
3. L.-J. Rong, Y.-T. Ye, X. Lin, X. Sun, S. Chen, J. Zhang and L. Zhang, *Dalton. Trans.*, 2024, **53**, 1947-1950.
4. S. T. Wang, Y. J. Liu, C. C. Feng, W. H. Fang and J. Zhang, *Aggregate*, 2022, **4**, e264.
5. D.-J. Li, Q.-h. Li, Z.-R. Wang, Z.-Z. Ma, Z.-G. Gu and J. Zhang, *J. Am. Chem. Soc.*, 2021, **143**, 17162-17169.
6. M.-Y. Gao, Z. Wang, Q.-H. Li, D. Li, Y. Sun, Y. H. Andaloussi, C. Ma, C. Deng, J. Zhang and L. Zhang, *J. Am. Chem. Soc.*, 2022, **144**, 8153-8161.
7. X. Fan, F. Yuan, D. Li, S. Chen, Z. Cheng, Z. Zhang, S. Xiang, S. Q. Zang, J. Zhang and L. Zhang, *Angew. Chem. Int. Ed.*, 2021, **60**, 12949-12954.
8.Y. Zhu, Z. Wang, D. Li, Y. D. Zhu, Q. H. Li, D. S. Li and L. Zhang, *Angew. Chem. Int. Ed.*, 2022, **61**, e202202853.
9.R. Xu, M. Zhu, W. Li, J. Ding and Y. Zhang, *The Journal of Physical Chemistry C*, 2023, **127**, 4258-4265.
10. C.-C. Feng, Z. Zhang, Z.-R. Wang, D.-J. Li, Q.-H. Li, L. Zhang and J. Zhang, *Inorg. Chem.*, 2021, **60**, 1885-1892.
11. B.-W. Xu, R.-J. Niu, Q. Liu, J.-Y. Yang, W.-H. Zhang and D. J. Young, *Dalton. Trans.*, 2020, **49**, 12622-12631.
12. C. Zheng, L. Lei, J. Huang, W. Chen, W. Li, H. Wang, L. Huang and D. Huang, *J. Mater. Chem. C*, 2017, **5**, 11579-11589.
13.Y. Zhu, Y. Su, Q. Zhou, P. Li, X.-Q. Wu, J. Zhao, W.-H. Fang, J. Zhang and D.-S. Li, *Nano Research*, 2025, **18**, 94907371.
14. J. B. Chen, F. Zou, P. P. Zhao, S. H. Shen, Y. H. Yu, H. X. Zhang and J. Zhang, *Angew. Chem. Int. Ed.*, 2025, **64**, e202519221.
15. P. Li, Q.-R. Sun, C.-B. Yu, J.-L. Chen, Y. Zhu, Q. Zhou, Z.-Y. Guo, S.-Y. Xiong, J.-H. Lv, T.-Y. Fu, C. Zheng, Q.-H. Li, W.-H. Fang, J. Zhang and D.-S. Li, *Chin. Chem. Lett.*, 2025, 111384.
16. J. Wu, D. liu, X. Yi and J. Zhang, *Science China Chemistry*, 2025, DOI: 10.1007/s11426-025-3162-9.
17. P. P. Zhao, X. M. Zhang, X. L. Zheng, Q. H. Li, H. X. Zhang and J. Zhang, *Small*, 2026, e14893.
